# Comparative proteomics reveals hydrogenase-centered steel-dependent growth and corrosion in *Desulfovibrio vulgaris* and *Desulfovibrio ferrophilus*

**DOI:** 10.3389/fmicb.2026.1944645

**Published:** 2026-09-22

**Authors:** Lipi Raghunatha Reddy, Nico Jehmlich, Annika Fiskal, Christian B. Fischer, Thomas Ternes, Jutta Meier, Arne Wick

**Affiliations:** 1 Department G2—Aquatic Chemistry, Federal Institute of Hydrology, Koblenz, Germany; 2 Department of Molecular Systems Biology, Helmholtz-Center for Environmental Research UFZ GmbH, Leipzig, Germany; 3 Department U2—Microbial Ecology, Federal Institute of Hydrology, Koblenz, Germany; 4 Department of Materials Science, Energy and Nano-engineering (MSN), College of Chemical Sciences and Engineering (CCSE), University Mohammed VI Polytechnic, Ben Guerir, Morocco; 5 Department of Biology, Institute for Integrated Natural Sciences, University of Koblenz, Koblenz, Germany

**Keywords:** biofilm, comparative proteomics, *Desulfovibrio ferrophilus*, *Desulfovibrio vulgaris*, FeS-mediated electron transfer, HIMET, microbially influenced corrosion, mild steel

## Abstract

Comparative proteomic analyses including ecologically distinct sulfate-reducing strains and different cellular fractions are scarce, limiting our understanding of corrosion-related mechanisms under anoxic conditions and the identification of protein signatures relevant to microbiologically influenced corrosion (MIC). To capture both freshwater and marine adaptations, we investigated the well-established SRB model strains *Desulfovibrio vulgaris* and *Desulfovibrio ferrophilus*. We compared energy metabolism, electron transport pathways, and growth patterns in both strains cultivated with lactate, H_2_, or steel as electron donor. Time-dependent biophysicochemical measurements, SEM/EDX, and shotgun proteomics were performed. We explored cellular proteins in planktonic and biofilm fractions, as well as potential extracellular enzymes in the supernatant. In both the strains, growth with steel revealed energy metabolisms more similar to H_2_-dependent metabolism instead of direct iron-to-microbe electron transfer (DIMET). Increased abundances of periplasmic hydrogenases and *c*-type cytochromes served as proteomic signatures supporting H_2_-mediated iron-to-microbe electron transfer (HIMET). Concomitant to bacterial growth and biofilm formation, mineral deposits of carbonates, phosphates and FeS were formed, the latter especially in saltwater treatments. The close association of *D. ferrophilus* cells with FeS particles supports the possibility of the role of FeS in electron transfer in addition to H_2_ as well as formate mediated electron transfer which may have contributed to the high corrosion levels with *D. ferrophilus*. Neither strain exhibited increased abundances of redox proteins known to be involved in extracellular electron transfer, supporting that DIMET may have not been a dominant mechanism under the given conditions. This interpretation is also in line with the observation that only a small proportion of the total cells were associated with the steel surface, and that direct cell-to-steel contact was not observed. These findings show that SRB can be metabolically flexible and that hydrogenase-based systems are sufficient for steel-dependent growth leading to corrosion. This is especially relevant in natural environments, where more than one type of electron donor is available and different modes of extracellular electron transfer exist.

## 1 Introduction

Microbially influenced corrosion (MIC) of steel structures in the environment is caused by different microbial groups, of which sulfate-reducing bacteria (SRB) are considered key contributors in anoxic marine and freshwater systems (Venzlaff et al., 2013; Tripathi et al., 2021; Fiskal et al., 2023; Reddy et al., 2025). SRB can utilize organic substrates, such as lactate and acetate, or inorganic substrates, such as H_2_ and even elemental iron, as electron donors to gain energy by dissimilatory sulfate reduction. The ability to use steel as an electron donor and the production of H_2_S, which is highly corrosive (Vita et al., 2015) mark SRB as a serious threat promoting localized corrosion to hydraulic metal structures, leading to large costs for the operation of river infrastructures (Binder and Graff, 1995; Kruger, 2011).

To understand the mechanisms by which SRB promote corrosion, it is essential to consider major electron-transfer pathways and the key steps of energy generation by dissimilatory sulfate reduction. In the cytoplasm, sulfate is first activated by sulfate adenylyltransferase (SAT) and then reduced stepwise to sulfide by adenylyl-sulfate reductase (AprAB) and dissimilatory sulfite reductase (DsrAB). Electrons derived from donor oxidation are transferred via cytochromes, menaquinone, ferredoxin, and DsrC, as well as membrane-bound complexes such as Qrc, Qmo, DsrMKJOP, Hmc, and Tmc, which support transmembrane electron transport. Energy conservation is further supported by membrane-bound complexes such as Ech, Coo and Rnf complexes, which contribute to the generation of electrochemical ion gradients that can be coupled to ATP synthesis (Pereira et al., 2011; Rabus et al., 2015).

Lactate is a common electron donor and carbon source for SRB (Vita et al., 2015), and in *Desulfovibrio* spp. it is typically incompletely oxidized to acetate (Kuever et al., 2015). Energy conservation during lactate oxidation involves both substrate-level and oxidative phosphorylation (Barton and Fauque, 2022). Electrons from lactate oxidation to pyruvate by lactate dehydrogenase (LDH) can enter the menaquinone pool, whereas pyruvate oxidation to acetyl-CoA by pyruvate:ferredoxin oxidoreductase (PFOR) generates reduced ferredoxin. Ferredoxin can be used by membrane-bound energy-conserving hydrogenases such as EchABCDEF and CooMKLXUHF, producing H_2_ that may be re-oxidized by periplasmic hydrogenases ([NiFe], [NiFeSe], and [FeFe]) in the hydrogen-cycling model (Odom and Peck, 1981; Matias et al., 2005; Pereira et al., 2011; Schölmerich and Müller, 2019). Periplasmic formate dehydrogenases may also contribute to redox balancing via CO_2_/formate interconversion. Finally, incomplete lactate oxidation ends with formation of acetate from acetyl-CoA via the phosphotransacetylase–acetate kinase pathway (Pta-Ack), with ATP generated through substrate-level phosphorylation by acetate kinase.

H_2_ is a ubiquitous electron donor readily consumed by SRB and is a favorable electron donor that results in high energy yield through sulfate reduction (Khosrovi and Miller, 1975; Tao et al., 2013; Massazza et al., 2021; Liu et al., 2025; Reddy et al., 2025). In the periplasm, H_2_ (endogenous and exogenous) is oxidized by periplasmic hydrogenases. The released electrons are transferred to periplasmic *c*-type cytochromes, e.g. *c3*, which deliver electrons to membrane-bound complexes (Qrc, Tmc, Hmc) that channel the electrons into the quinone pool or transfer them across the membrane to cytoplasmic carriers. Electrons are passed from the quinone pool via membrane-bound energy-conserving complexes (QmoABC, DsrMKJOP) for sulfate reduction (Pereira et al., 2011; Rabus et al., 2015). Alternatively, Ech and Coo can work in reverse under H_2_-rich conditions by oxidizing H_2_ to regenerate reduced ferredoxin, supporting intracellular redox balance.

Reduced ferredoxin is central to redox balance and biosynthesis (Wenk et al., 2018; Fuchs, 2022). However, many electron donors do not have a sufficiently low redox potential to reduce it directly; instead, electron flow is often mediated by flavin-based electron bifurcation (FBEB). FBEB couples exergonic and endergonic reactions via soluble flavin-dependent complexes. Such protein complexes involved in FBEB have been identified in SRB, including HdrABC/MvhADG-like complexes and FlxABCD–HdrABC systems (Rabus et al., 2015).

Steel can also serve as an electron donor (Dinh et al., 2004; Kato, 2016; Gupta et al., 2020; Lovley, 2022). Owing to its insoluble nature, microorganisms require extracellular electron transfer mechanisms to extract electrons from the metal surface (Gupta et al., 2020; Lekbach et al., 2021). Direct iron-to-microbe electron transfer (DIMET)-capable microorganisms are generally considered to have a high corrosion potential and electron transfer may occur via outer membrane multiheme cytochromes (OMCs) (Dinh et al., 2004; Chatterjee et al., 2021; Lahme et al., 2025). Alternatively, electrons may be acquired via H_2_-mediated iron-to-microbe electron transfer (HIMET). Under certain conditions, abiotic H_2_ formation at steel surfaces may be influenced by extracellular hydrogenases or FeS phases (Deng et al., 2020; Lekbach et al., 2021; Woodard et al., 2023), although sulfide can also limit H_2_ release by forming passivating FeS layers (Ma et al., 2000; Luo et al., 2018). H_2_ forms abiotically through the reaction of sulfide with Fe^0^ to produce FeS (Venzlaff et al., 2013; Deutzmann et al., 2015; Woodard et al., 2023). Formate-mediated iron-to-microbe electron transfer is another possibility, in which formate forms abiotically on steel via CO_2_ reduction, potentially catalyzed by extracellular formate dehydrogenases (Lekbach et al., 2021).

DIMET vs. HIMET in *Desulfovibrio* spp. remains debated (Liang et al., 2021; Woodard et al., 2023; Li et al., 2025). Details of these studies are provided by Reddy et al. (2025). Proteomics studies suggest that outer membrane multiheme cytochromes are associated with DIMET in *D. ferrophilus*, whereas [FeFe] and [NiFe] hydrogenases have been linked to HIMET in other studies (Dinh et al., 2004; Beese et al., 2013; McCully and Spormann, 2020; Chatterjee et al., 2021; Liang et al., 2021; Lovley, 2022; Ueki and Lovley, 2022; Li et al., 2025). Apart from HIMET and DIMET, the third proposed mechanism is the shuttle-mediated iron-to-microbe electron transfer (SIMET), which involves extracellular redox-active molecules; however, there is little evidence for this mechanism in *Desulfovibrio* spp. (Lekbach et al., 2021; Woodard et al., 2023).

In summary, most pure-culture studies with SRB do not fully exclude H_2_-mediated electron transfer (HIMET), whereas conclusive experimental evidence for direct electron transfer (DIMET) remains limited. Only a few studies have examined protein-level responses by comparing three electron donors while distinguishing between planktonic and biofilm fractions. In this study, we compare the growth characteristics and energy metabolism of the two corrosive *Desulfovibrio* strains, *D. vulgaris* (freshwater) and *D. ferrophilus* (saltwater). We use comparative proteomics to assess protein-level regulation with lactate, H_2_, and steel as electron donors and to explore potential corrosion mechanisms. Time-dependent biophysicochemical measurements were used to distinguish biotic from abiotic processes and quantify the contribution of MIC. Scanning electron microscopy (SEM) coupled with energy-dispersive X-ray (EDX) spectroscopy was used to characterize steel surface morphology, biofilm formation, and cell–mineral interactions. Proteomic analyses were performed on supernatant and cellular (planktonic and biofilm) fractions. Biofilm cells may exhibit protein profiles associated with surface attachment and DIMET, whereas planktonic cells may rely on HIMET or SIMET; in this case, similarities in the protein profiles of the cells grown on steel or H_2_ may be expected. Supernatants may contain extracellular enzymes, such as hydrogenases, that facilitate abiotic H_2_ formation. By comparing proteomes across fractions and electron donors, we aim to identify marker proteins and pathways relevant to natural corrosion sites and to assess whether the organisms employ specialized steel-adapted electron-transfer systems or their existing hydrogenase-based metabolism to access electrons derived from steel oxidation.

## 2 Materials and methods

### 2.1 Bacterial strains and media preparation

Experiments were performed with *D. vulgaris* DSM 2119 and *D. ferrophilus* DSM 15579 obtained from the Leibniz Institute DSMZ—German Collection of Microorganisms and Cell Cultures GmbH, Braunschweig, Germany. The freshwater medium was prepared based on Widdel and Bak (1992) with slight modifications (see Supplementary Table S1), and the saltwater medium corresponded to the DSMZ medium 195c for *Desulfobacter* sp. (Koblitz et al., 2023). Both media were buffered with NaHCO_3_ (30 mM final concentration). Sulfate was provided as the electron acceptor by adding Na_2_SO_4_ with a final concentration of 20 mM. For lactate treatments, Na-L-lactate was provided at a final concentration of 45 mM. In the steel and H_2_ treatments (see Table 1), the organic C-source was supplemented as Na-acetate with a final concentration of 1 mM. The reducing agent Na_2_S·9H_2_O was provided only for the saltwater medium (1.69 mM final concentration). All stock solutions were autoclaved (121 °C for 20 min), except the vitamin solutions were 0.2-μm filter-sterilized. The pH was adjusted between 7.0–7.4 by addition of sterile 1 M HCl.

**Table 1:** Composition of the eight treatments.

Code names	Strain	Media type	Electron donor			Additional organic C
			Lactate (45 mM)	H_2_ (80% sat.)	Steel	Acetate (1 mM)
Dv-L	*D. vulgaris* DSM 2119	Freshwater	x			
Dv-H_2_				x		x
Dv-St					x	x
Df-L	*D. ferrophilus* DSM15579	Saltwater	x			
Df-H_2_				x		x
Df-St					x	x
Ab-fresh		Freshwater			x	x
Ab-salt		Saltwater			x	x

Pre-cultures for inoculum were set up from glycerol stocks; 1.5 ml were thawed and transferred to 50 ml of the corresponding medium with lactate in sterile 120-ml serum bottles. The bottles were sealed with autoclaved butyl rubber stoppers and aluminum crimps. The headspace was flushed with 80% N_2_ (5.0)/20% CO_2_ (4.8). The inoculated bottles were incubated at 28 °C on a horizontal shaker at 110 rpm, until stationary phase (determined by whole-cell protein using Bradford assay). After seven days, the pre-culture was transferred to 50-ml sterile centrifuge tubes and centrifuged at 4,863 x *g* and 20 °C for 30 min. The supernatant was discarded, and the cell pellets were washed with 5 ml of the corresponding mineral medium for the removal of residual lactate, free sulfides and other soluble metabolites and medium components. The washed cell pellets were resuspended in 50 ml respective mineral medium for the final inoculation (final total protein concentration of 6 mg l^−1^) of the different treatments of this study. Media preparation and all handling of stocks and samples (see below) were done under Ar (5.0) atmosphere in an anaerobic chamber (MEGA E-Line, M-Line, GS Glovebox Systemtechnik Malsch, Germany).

### 2.2 Experimental set-up and sample collection

In total, eight different treatments were applied and performed in triplicate (Table 1). The respective triplicates are indicated as ‘a', ‘b' and ‘c'. The growth of the two strains were investigated in the presence of the three electron donors: lactate (45 mM), H_2_ (80% in the headspace corresponding to 49.05 mM in 100 ml medium) and Fe^0^. In addition, the abiotic corrosion in the two media were tested. Fe^0^ was provided as high-strength low-alloy structural steel type S355J2 (ASTM A572, Gebr. Recknagel Präz. GmbH, Christes, Germany) as coupons in the size of 7.6 x 1.0 x 0.2 cm^3^ and as small platelets in the size of 1.0 x 1.0 x 0.2 cm^3^ mounted on polycarbonate coupons (PC) for SEM analysis (for details see Reddy et al., 2025). The coupons and platelets were cleaned by immersion in ≥ 96% denatured ethanol for 30 min and dried with N_2_ (5.0). One steel coupon and one PC-mounted platelet were placed each into a 250-ml sterile serum bottle for the biotic treatments or a 120-ml sterile bottle for the abiotic treatments. The aluminum foil covered bottles were heat sterilized at 120 °C twice consecutively for 5 h. According to the treatments, 100 ml of the respective medium was dispensed into sterile serum bottles for the biotic and 80 ml for the abiotic treatments. The biotic treatments were inoculated with the prepared cell suspension. The bottles were sealed, and the headspace was flushed with 80% N_2_ (5.0)/20% CO_2_ (4.8) for lactate and steel treatments and with 80% H_2_ (5.0)/20% CO_2_ (4.8) for H_2_ treatments. The inoculated bottles were incubated at 28 °C on a horizontal shaker at 110 rpm for 17 days (lactate and H_2_ treatments) and 31 days (steel treatments). The incubation was terminated when sulfate concentrations stopped decreasing in the biotic steel treatments, or when the lactate and H_2_ concentrations were below the detection limit in the corresponding biotic treatments.

The first sampling took place on day 0 (directly after inoculation) and subsequent samplings on a weekly basis for all treatments except the abiotic steel treatments, which were sampled at indicated intervals (Supplementary Table S5). Prior to sampling, the headspace was brought to normal pressure by compensation with Ar. Samples from the headspace were collected for the detection of hydrogen gas in the H_2_ and steel treatments (biotic and abiotic). For this purpose, 1 ml was withdrawn and injected into 4-ml vials (Bottles G4, DIN ISO 719; Klaus Ziemer, Langerwehe, Germany) and stored at room temperature. The sampling of liquid samples was done through injection of 5 ml of the respective sterile medium followed by thorough shaking and withdrawal of 5 ml culture liquid, which was transferred into sterile 15-ml centrifugation tubes. The liquid was analyzed for pH, free sulfide, Fe(II), lactate, acetate, formate, sulfate, *dsrAB* gene copy number and whole-cell protein. On the final day, samples from the culture liquid, along with biofilm and corrosion products scraped off from the steel coupons and resuspended in 2 ml sterile mineral medium, were harvested for proteomics (biotic treatments only). After removal of biofilm and corrosion products, the steel coupons were determined for weight loss. For the coupons in saltwater treatments, the total amount of Na_2_S (initial amount plus the amount added at each sampling) was calculated, and the corresponding iron loss due to this cumulative S^2−^ addition was determined. This loss was then subtracted from the total coupon weight loss to estimate the weight loss attributable to abiotic H^+^ reduction and MIC (see details in Supplementary Tables S5, S6).

### 2.3 Monitoring of the biophysicochemical trends and SEM/EDX analysis of biofilm and steel coupon

The measurement of H_2_ in the gas phase was performed by a Unisense NP sensor (H_2_-X-NP-103803, Unisense, Aarhus, Denmark, details in section 1.1, Supplementary file 1). Measurements of pH were performed directly after sampling with a pH-meter and pH-electrode HA405-DPA-SC-S8/225 (both from Mettler-Toledo AG, Schwerzenbach, Switzerland). HCl-soluble Fe(II) was determined photometrically on fresh samples according to Lovley and Phillips (1987) using the ferrozine method (Stookey, 1970). Free sulfide was determined on fresh samples using the photometric method of Cline (1969). Whole cell protein was determined for pellets obtained from 1 ml liquid sample by Bradford assay (Bio-Rad Protein Assay Dye Reagent Concentrate, Bio-Rad Laboratories GmbH, Feldkirchen, Germany). Concentrations of sulfate and organic acids (acetate, lactate and formate) were measured in the supernatants by ion chromatography with a Metrosep A Supp 19–250/4.0 column (Metrohm, Herisau, Switzerland). The scraped-off steel coupons were immersed for 5 min in inhibited HCl to remove all corrosion products, and weight loss (mg cm^−2^ and mmol l^−1^) was determined. The PC-mounted steel platelets were prepared for SEM/EDX analysis as described in Reddy et al. (2025). The platelets were mounted on stubs with double-sided adhesive carbon pads and were sputter-coated with 15 nm Au. SEM images were recorded with a field emission scanning electron microscope JSM-IT800 (HL, JEOL Ltd., Tokyo, Japan) at accelerating voltages of 2 kV using standard SE detector. Energy-dispersive X-ray spectroscopy and chemical element mapping were performed with a silicon drift detector (SDD) EX-74710U1L4Q (DrySD^TM^60 Detector, JEOL Ltd.) at accelerating voltages of 15 kV.

### 2.4 DNA extraction and droplet digital PCR (ddPCR)

DNA was extracted from 1 ml of the liquid sample and 1 ml of the biofilm slurry using the DNeasy Blood and Tissue Kit (Qiagen, Hilden, Germany). DNA was eluted in 100 μl of DNase-free buffer. Absolute abundance of the *dsrAB* genes from the extracted DNA was determined by ddPCR using the primer set DSR-1F+ 5′-ACSCACTGGAAGCACGCCGG-3′ and DSR-R 5′-GTGGMRCCGTGCAKRTTGG-3′ (expected product size 221 bp, details in section 1.2, Supplementary file 1). Gene abundance (copies μl^−1^) was calculated for the initial dilution of the DNA template and the dilution of the reaction volume during droplet generation. The final gene copies were represented per milliliter of culture.

### 2.5 Statistical tests

Statistical significance test of the biophysicochemical parameters, growth yields and steel weight loss between the treatments was performed by Student's *t*-test and one-way ANOVA using SigmaPlot 13.0 (Inpixon GmbH, CA, USA) software.

### 2.6 Metaproteomics analysis

#### 2.6.1 Proteomics analysis of the planktonic, biofilm and supernatant samples

Proteins from spent culture liquid (supernatant), planktonic and biofilm cells were analyzed by bottom-up proteomics as described in detail in section 1.3, Supplementary file 1. Proteomics analysis was performed separately for each treatment replicate. For each replicate, 100 ml of culture liquid and 1 ml of biofilm slurry were processed to separate cell pellets from the corresponding supernatants. Cell lysates from planktonic and biofilm pellets were prepared by mechanical bead beating followed by ultrasonication according to Reddy et al. (2025). Instead of the reported 1.25% SDS buffer, SDT buffer was used, consisting of 1% sodium dodecyl sulfate (SDS) and 5 mM dithiothreitol in 0.1 M tris(hydroxymethyl)aminomethane hydrochloride (Carl Roth, Karlsruhe, Germany). To enrich proteins from the supernatant fractions, samples were concentrated using 10 kDa molecular weight cutoff filters (MWCO, Amicon Ultra-15 and Ultra-0.5 centrifugal filters, Merck Millipore, Molsheim, France) according to the manufacturer's instructions. Concentrated proteins were subsequently precipitated with ice-cold acetone, and the resulting protein pellets were resolubilized in 100 μl of SDT buffer. Aliquots of 30–60 μl from cell lysates and resolubilized supernatant proteins were subjected to reduction, alkylation and overnight tryptic digestion at 37 °C. Peptides were desalted using C_18_ ZipTip columns (Merck Millipore Molsheim, France) dissolved in 0.1% formic acid (Jehmlich et al., 2014), and analyzed by nanoliquid chromatography mass spectrometry (UltiMate 3000 RSLCnano, Dionex, Thermo Fisher Scientific). Eluted peptide lysates were subsequently analyzed using a Q Exactive HF mass spectrometer (Thermo Fisher Scientific) coupled with a TriVersa NanoMate (Advion, Ltd., Harlow, UK) as described in section 1.3.3, Supplementary file 1.

#### 2.6.2 Proteomic data evaluation

MS data were evaluated using Proteome Discoverer v2.5 (Thermo Fisher Scientific). Reference ^*^.fasta databases for *D. vulgaris* and *D. ferrophilus* were obtained from NCBI (https://www.ncbi.nlm.nih.gov/nuccore). In addition, common contaminant proteins from the common Repository of Adventitious Proteins database (cRAP; 116 entries, https://www.thegpm.org/crap/) were included. The processing workflow followed the settings described by Reddy et al. (2025). The complete protein list generated in this study has been deposited in the Figshare repository and is available at: [https://doi.org/10.6084/m9.figshare.33005042]. Protein identifications assigned to taxa other than *D. vulgaris* or *D. ferrophilus* were excluded from downstream analyses, as the experiment was performed with pure cultures of the respective organisms. Proteins that differed significantly in abundance (|log_2_FC| ≥ 2 and p-value ≤ 0.05) were grouped according to putative functions in each of the treatments following the nomenclature planktonic (pl), biofilm (bf), culture supernatant (su), and biofilm supernatant (bf-su). Non-metric multidimensional scaling (NMDS) was performed in R (v. 4.6.0) using the vegan package (v. 2.7-5) based on Bray-Curtis dissimilarities. The ordination plot was generated using ggplot2 (v. 4.0.3, Wickham, 2016; Oksanen et al., 2026). Heatmaps were generated in R using the ComplexHeatmap package (v. 2.28.0), with color mapping implemented using the circlize package (v. 0.4.18, Gu et al., 2014, 2016; Gu, 2022).

## 3 Results

### 3.1 Biophysicochemical trends and electron balances

The biophysicochemical trends monitored for *D. vulgaris* and *D. ferrophilus* throughout the incubation period are depicted in Figure 1. No activity was observed in the third replicate of the lactate treatment of *D. ferrophilus* (Df-L-c), henceforth, the values were not included. The rise in pH supported the establishment of a reducing environment in the biotic treatments (Supplementary Figures S1A, B). The electron donors lactate and H_2_ were not detectable anymore after day seven for *D. vulgaris* (Dv-L, Dv-H_2_; Figures 1A, B) and after day 10 for *D. ferrophilus* (Df-L, Df-H_2_; Figures 1D, E). A concomitant production of acetate as the metabolic end-product of incomplete lactate oxidation was observed for both strains (Dv-L, Df-L; Figures 1C, F). A small increase in acetate also occurred in Dv-H_2_. In the lactate treatments, based on the acetate produced, about 85–88% of the lactate consumed was utilized for energy and about 15–12% for biomass in both strains (Dv-L, Df-L). Corresponding to lactate and H_2_ consumption, sulfate, as an electron acceptor, decreased (Figures 1G, J), accompanied by a rise in sulfide (Figures 1H, K). The electron balances of lactate or H_2_ oxidation vs. sulfate reduction agreed well for Dv-L, Df-L, and Df-H_2_ (Table 2), except Dv-H_2_ showed a lower value, indicating that the measured H_2_ consumption was not completely consistent with the amount of sulfate reduction.

**Figure 1:**
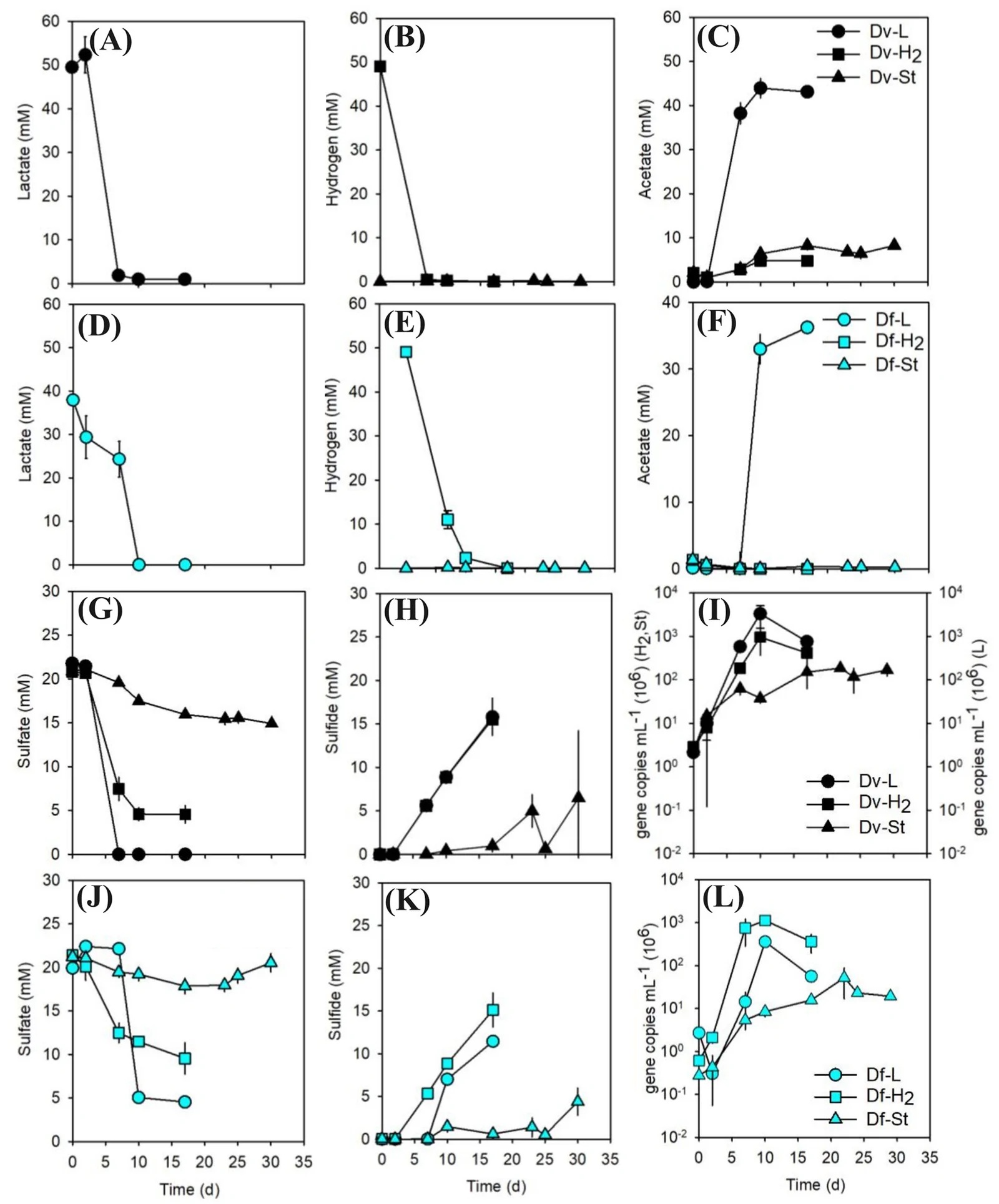
Time trends of the biophysicochemical parameters lactate **(A, D)**; H_2_ **(B, E)**; acetate **(C, F)**; sulfate **(G, J)**; sulfide **(H, K)** and *dsrAB* copy numbers **(I, L)** monitored for *D. vulgaris* **(A, B, C, G, H, I)** and *D. ferrop*hilus **(D, E, F, J, K, L)** over 17 days (lactate and H_2_ treatment) and 30 days (steel treatments) of incubation. Error bars indicate standard deviations of the triplicates except for Df-L treatments which includes the mean of only two replicates as the third replicate (Df-L-c) did not show growth before day 17.

**Table 2:** Changes in concentrations (Δ), growth parameters and calculated electron balances.

Parameters	Dv-L	Dv-H_2_	Dv-St	Df-L	Df-H_2_	Df-St	Ab-fresh	Ab-salt
Δ Lactate (mM)	−48.5 ± 0.87	N.A.	N.A.	−41.5 ± 0.57	N.A.	N.A.	N.A.	N.A.
Δ Acetate (mM)	43.10 ± 0.05	2.73 ± 0.25	6.2 ± 1.2	32.2 ± 0.43	−1.36 ± 0.23	−1.10 ± 0.24	1.29 ± 0.03	1.38 ± 0.16
Δ Formate (mM)	0.09 ± 0.02	−0.06 ± 0.01	−0.06 ± 0.00	0.04± 0.01	−0.06 ± 0.01	−0.06 ± 0.00	0.50 ± 0.18	0.42 ± 0.14
Δ H_2_ (mM)	N.A.	−49.05 ± 0.00	N.A.	N.A.	−49.05 ± 0.00	N.A.	2.24 ± 0.45	1.00 ± 0.05
Fe^0^ decrease (mM)	N.A.	N.A.	5.09 ± 0.21	N.A.	N.A.	8.8 ± 1.0 (6.5 ± 1.3)^**#**^	3.36 ± 0.76	3.70 ± 0.05 (1.60± 0.056)^#^
Δ ${\text{SO}}_{4}^{2-}$ (mM)	−23.5 ± 0.53	−16.2 ± 0.87	−5.96 ± 0.08	−16.3 ± 0.49	−11.9 ± 1.6	−3.36 ± 0.77	1.3 ± 0.79	1.8 ± 0.81
SRR^y^ (mmol ${\text{SO}}_{4}^{2-}$ l^−1^ d^−1^)	1.28 ± 0.03	0.95 ± 0.05	0.29 ± 0.02	0.96 ± 0.03	0.67 ± 0.09	0.22 ± 0.05	N.A.	N.A.
Electron balance (donor/acceptor) (%)	103.5 ± 0.55	75.8 ± 4.1	21.4 ± 0.80	127.3 ± 2.1	94.82 ± 0.68	67.1 ± 8.1	148.9 ± 5.4	371 ± 21 (160.33 ± 11.16)^#^
Total cell protein (mg l^−1^)	466 ± 24	96 ± 17	27.9 ± 3.9	392.21^$^	265.5 ± 3.5	5.6 ± 0.41	N.A.	N.A.
Planktonic *dsrAB* copies (10^6^ ml^−1^ culture)	760 ± 150	420 ± 350	170 ± 48	56.1 ± 2.5	360 ± 160	19.2 ± 2.2	N.A.	N.A.
Biofilm *dsrAB* copies (10^6^ ml^−1^ culture)	N.A.	N.A.	0.51 ± 0.31	N.A.	N.A.	0.60 ± 0.27	N.A.	N.A.

The values represented with a minus sign indicate a decrease in concentration. N.A. stands for ‘not applicable'.

The values of Df-L treatments include the mean of only two replicates, as the third replicate (Df-L-c) did not show growth before day 17.

^y^Sulfate reduction rates (SRR) were calculated for all treatments for the first 17 days of incubation because lactate and H_2_ treatments had no further decrease in sulfate concentration beyond day 17.

^#^Steel weight loss after subtracting weight loss due to added Na_2_S.

^$^Due to slow growth, the incubation period of replicate Df-L-a was extended to 30 days. The reported protein value for Df-L corresponds to this replicate.

Contrary to treatments with lactate and H_2_, the biophysicochemical changes in biotic treatments with steel as an electron donor (Dv-St, Df-St) were more gradual. A significantly higher steel weight loss occurred in Dv-St and Df-St compared to the corresponding abiotic steel treatments (*p* < 0.05, Ab-fresh, Ab-salt, Figure 2A, Table 2). Upon subtraction of the weight loss due to the added Na_2_S (2.25 mM, 26% of total averaged steel weight loss in Df-St treatments), the Df-St weight loss was still higher but not significantly (*p* > 0.05) compared to Dv-St. Sulfate decrease in the steel treatments was slow compared to the respective counterpart treatments with lactate or H_2_ (Figures 1G, J; see also sulfate reduction rates in Table 2). As a result of iron sulfide precipitation, the overall concentration of free sulfide was lower compared to treatments with lactate and H_2_ but higher compared to the abiotic ones (Supplementary Figures S2C, D). Exceptionally high values detected in Dv-St might have been due to small FeS particles in suspension that might have interfered with the sulfide determination assay (Figures 1H, K). The result of sulfide precipitation was accompanied by a rise in Fe(II) precipitates, especially in Df-St (Supplementary Figure S1C). Both the strains reflected high measured concentrations of Fe(II) precipitates (Fe_s_) and sulfides compared to the abiotic ones (Supplementary Figures S2A, C, D). No H_2_ evolution was observed with biotic steel treatments (compared to the abiotic ones), indicating that it was consumed by the bacteria (Figures 1B, E, Table 2). In terms of the electron balance, only 21% of the electrons released from steel weight loss corresponded to the sulfate reduced in Dv-St. In Df-St, an equivalent of 67% was observed (Table 2). A higher sulfate decrease was observed in Dv-St compared to Df-St (*p* > 0.05), in contrast to the steel weight loss pattern. Poor electron balances might be due to the underestimation of steel weight loss and/or the overestimation of the amount of sulfate reduced. The latter was derived based on the amount of sulfate removed, which may also include sulfate (co)precipitated with Fe carbonates or phosphates. Similar to Dv-H_2_ but more pronounced, an increase in acetate occurred in Dv-St (Figure 1C, Table 2).

**Figure 2:**
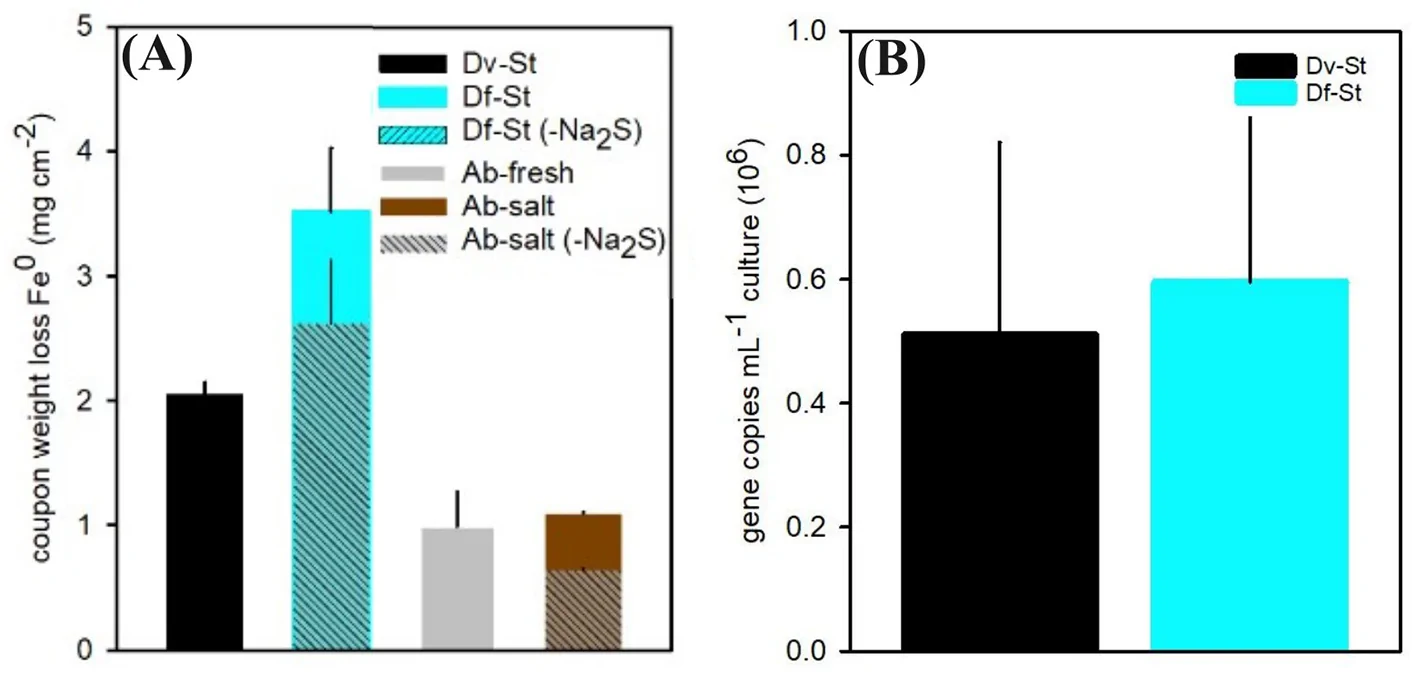
**(A)** Averaged steel weight loss per surface area from treatments (Dv-St, Df-St) and from abiotic treatments (Ab-fresh, Ab-salt). Additional bars for Df-St and Ab-salt mark weight losses after subtraction of weight losses by added Na_2_S (Df-St (-Na_2_S), Ab-salt (-Na_2_S)). **(B)** Averaged *dsrAB* gene copy number of the biofilm samples from Dv-St and Df-St determined on the last day of incubation (day 30).

In the abiotic steel treatments, little change in sulfate concentrations was observed (Supplementary Figure S2B), and the steel weight losses were similar between Ab-fresh and Ab-salt (Figure 2A, Table 2). The addition of Na_2_S (2.10 mM) accounted for 57% of the total averaged steel weight loss in Ab-salt. H_2_ evolution was observed in both the abiotic steel treatments; however, it was most likely underestimated, as the headspace was sparged after the addition of the medium and its first reaction with the steel. This may have been responsible for the poor electron balances (Table 2). This is especially true for Ab-salt, where the medium contained Na_2_S which most likely immediately reacted with Fe^0^. The formation of acetate and formate was also observed for both abiotic treatments (Table 2).

### 3.2 Growth trends with lactate, H_2_ and steel as electron donors

Based on the available electron donors and C-sources, different growth and energy conservation patterns in the two strains can be inferred. Chemoorganoheterotrophic growth in the lactate treatments plausibly allowed the highest growth yields, as indicated by *dsrAB* copy numbers and whole-cell protein (Table 2). Within the H_2_ and steel treatments, with CO_2_ in the gas phase and 1 mM acetate as C-sources, chemolithoheterotrophic as well as chemolithoautotrophic growth was possible. Particularly, *D. vulgaris* in steel and H_2_ treatments apparently grew autotrophically, as unexpected accumulation of acetate was observed (Figure 1C; Table 2). Whereas *D. ferrophilus* in H_2_ and steel treatments, acetate was consumed as anticipated, indicating heterotrophic growth (Figure 1F; Table 2). The planktonic growth yield for both strains was observed to be the second highest in the H_2_ treatments, followed by the steel treatments (Table 2). The planktonic *dsrAB* copy numbers were much higher than the biofilm gene *dsrAB* numbers for both strains (Figure 2B, Table 2). The biofilm *dsrAB* copy numbers of *D. ferrophilus* were higher than those of *D. vulgaris*; however, the difference was not significant (*p* > 0.05). Correspondingly, a higher percentage of *D. ferrophilus* cells (~3 % of the total cells) were found in the biofilm compared to *D. vulgaris* (~0.3% of the total cells).

### 3.3 Observation of biofilm and corrosion products on steel by SEM/EDX analysis

Distinct mineral morphologies were observed across all steel treatments. Large slab-like structures were enriched in O, Fe, C, and P, with minor Mg signals, suggesting the presence of iron phosphate phases [indicated in Figures 3A(i), 4A(i)]. Cubic precipitates showed enrichment in O, Fe, and C, with elevated Ca and minimal or no detectable P, consistent with Ca/Fe carbonate phases [indicated in Figures 3D(i), 4A(i), B]. Flake-like structures were predominantly enriched in Fe and S, indicative of iron sulfide formation (Figures 3, 4, indicated in red and marked with red arrows). Representative EDX data are provided in Supplementary Table S1.1, Table 1.

**Figure 3:**
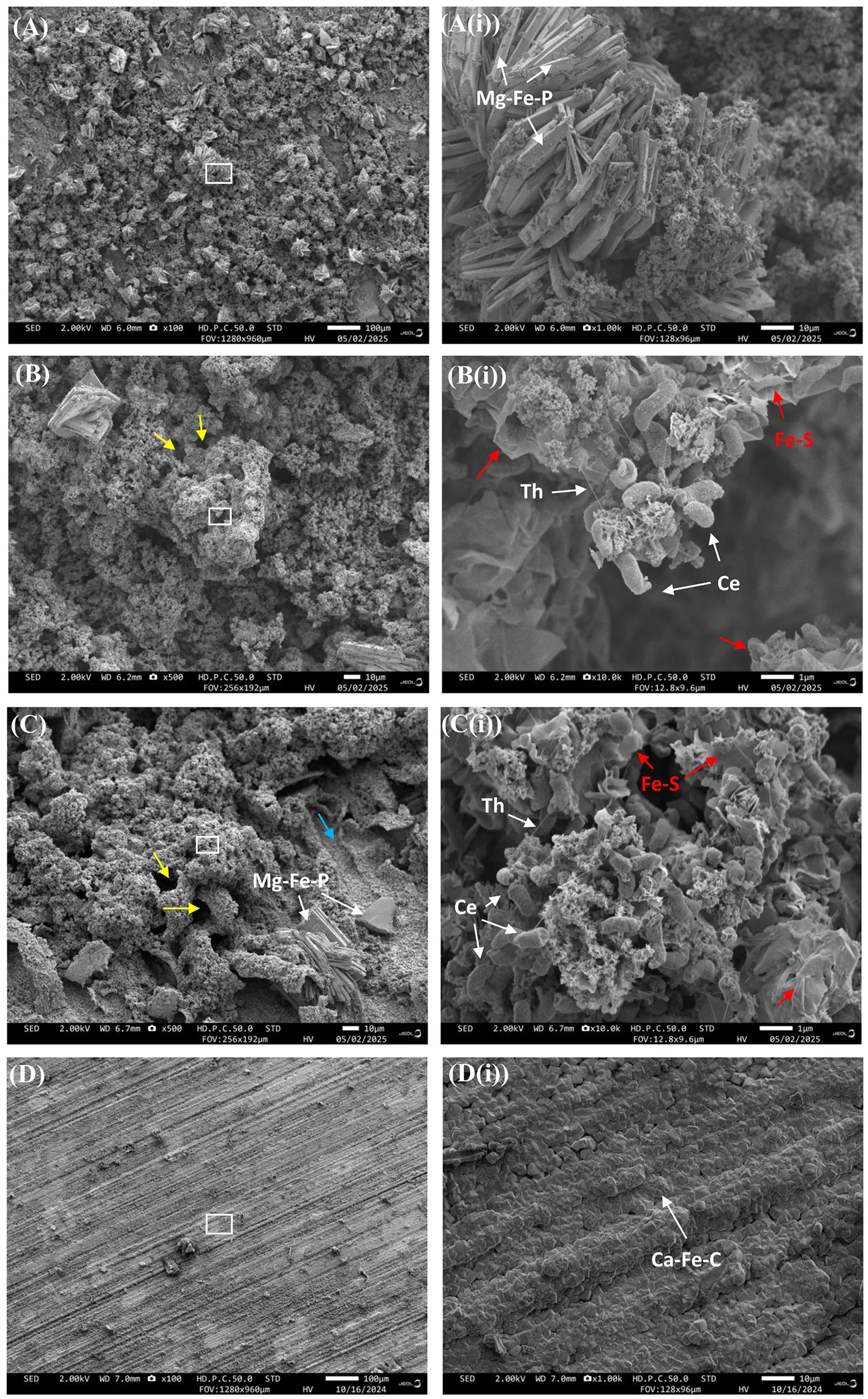
SEM images of the steel platelets in treatments with *D. vulgaris* **(A–C)** and abiotic freshwater treatments **(D)** after 30 days of incubation. Magnification of images were 100x **(A, D)**; 500x **(B, C)**; 1000x **(A(i), D(i))** and 10,000x **(B(i), C(i))**. The panels labeled with **(i)** represent a magnified image of the area marked by a white rectangle in the image with the corresponding letter. The images were recorded with a SE detector at 2 kV. Images **C, C(i)** were recorded at a tilt of 45°. Th: thread-like structures; Ce: cells; Mg-Fe-P: iron phosphates; Fe-S: iron sulfides; Ca-Fe-C: iron/calcium carbonates; yellow arrows: hollow cavities; red arrows: flaky iron sulfides; blue arrow: biofilms closer to the steel surface.

**Figure 4:**
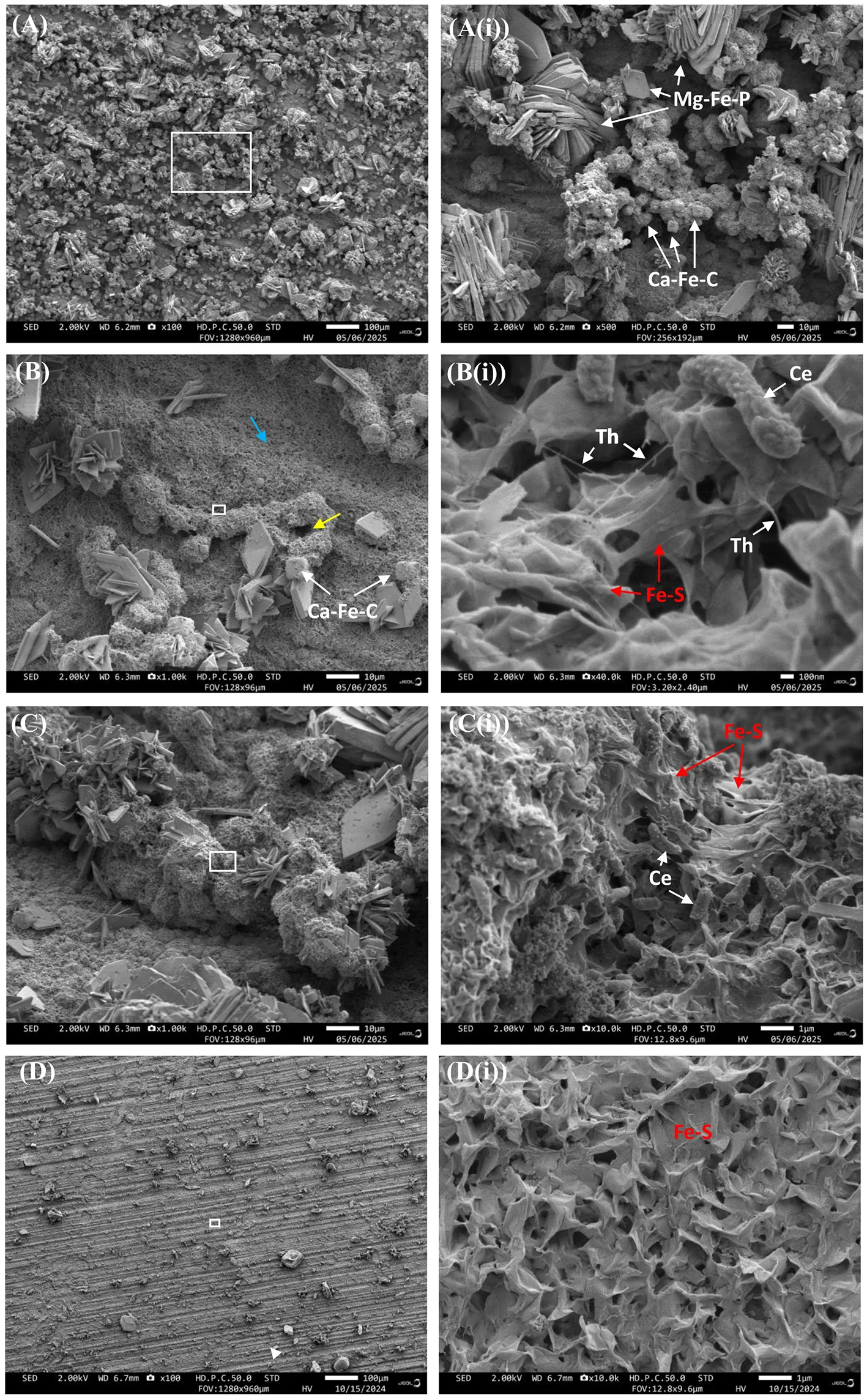
SEM images of the steel platelets in treatments with *D. ferrophilus* **(A–C)** and abiotic saltwater treatments **(D)** after 30 days of incubation. Magnification of images were 100x **(A, D)**; 500x **[A(i)]**, 1000x **(B, C)**; 10,000x **[C(i), D(i)]**; and 40,000x **[B(i)]**. The panels label*l*ed with (i) represent a magnified image of the area marked by a white rectangle in the image with the corresponding letter. The images were recorded with an SE detector at 2 kV. Images **C, C(i)**, were recorded at a tilt of 45°. Th: thread-like structures; Ce: cells; Mg-Fe-P: iron phosphates; Fe-S: iron sulfides; Ca-Fe-C: iron/calcium carbonates; yellow arrows: hollow cavities; red arrows: flakey iron sulfides; blue arrow: biofilms closer to the steel surface.

*D. vulgaris* (Dv-St) formed strikingly thick and structurally heterogeneous biofilms (estimated height ~736 μm) with the vibrio-shaped cells embedded within a dense mineral matrix containing maybe also polysaccharides or other biopolymers (Figures 3A–C). Minerals in the vicinity of the cells often showed flake-like morphologies, consistent with iron sulfide precipitation (Figures 3B, C, Supplementary Table S1.1). Additionally, cells were frequently located along protruding mineral edges and appeared to be connected by filamentous structures stretched between the cells and mineral surfaces [Figures 3B(i), C(i)]. These filaments may represent extracellular polymeric substances (probably formed by dehydration during sample preparation) or even fine mineral structures. Next to high-piled mineral-biofilm agglomerates producing many hollow cavities (Figures 3B, C, yellow arrows), biofilms were also observed to form on steel surfaces (Figure 3C, blue arrow), where cells were equally embedded within a mineral-rich matrix.

The steel surface in treatments with *D. ferrophilus* (Df-St) showed an apparently similar abundance of larger mineral precipitates (Figure 4A). However, at higher magnification it can be seen that the biofilm consists mainly of flakey, partially very thin iron sulfides, with an estimated layer height of ~78 μm [Figures 4B(i)–C(i), Supplementary Table S1.1] that were very similar in morphology to those of the abiotic saltwater treatment [Ab-salt, Figure 4D(i)]. Again, thin thread-like structures (indicated as Th) were observed, which could result from extracellular polymers or even resulting from iron sulfide precipitates [Figure 4B(i)]. The vibrio-shaped cells embedded within the mineral matrix were more frequently concealed and less often found on top of the mineral surfaces compared to *D. vulgaris* [Figures 4B(i)–C(i)]. In addition, biofilms of *D. ferrophilus*, which occurred closer to the steel surface, were more frequently observed in addition to mineral-biofilm agglomerates (e.g., Figure 4B, blue arrow).

Contrary to the biotic treatments, the abiotic treatments showed a comparatively smoother surface morphology without prominent mineral agglomerates, and the original steel topography was still visible (Figures 3D, 4D). In the Ab-fresh treatments, the surface was primarily covered by a carbonate-rich corrosion crust [Figure 3D(i), Supplementary Table S1.1]. Whereas, in the Ab-salt treatments, flaky iron sulfide precipitates derived from the medium covered the whole surface [Figure 4D(i), Supplementary Table S1.1]. In addition, for both biotic and abiotic treatments, SEM/EDX analysis of the underlying steel surfaces following corrosion products and biofilm removal revealed no detectable pitting or cracking (Supplementary Figures S3A–H).

### 3.4 Differential proteomic analysis

#### 3.4.1 General aspects of the protein profiles

In total, about 6,000 proteins were identified across all samples (see 2.6.2 for complete protein list information). The proteins selected for in-depth analysis were related to energy metabolism and corrosion, including those for dissimilatory sulfate reduction; lactate metabolism; H_2_-associated hydrogenases ([NiFe]/[FeFe] hydrogenases); formate dehydrogenases; cytochromes; Ech complexes; Rnf complexes; potential FBEB; ATP synthases; cytoplasmic transporters; outer membrane proteins; potential biofilm formation/regulation and flagellar/motility (Supplementary Table S2.1, Table 2). Upon screening the proteins, the categories that we found relevant to growth with steel and H_2_ (H_2_-associated hydrogenases ([NiFe]/[FeFe] hydrogenases); formate dehydrogenases; cytochromes; Ech complexes; Rnf complexes; potential FBEB; outer membrane proteins) along with the category ATP synthases are included in Figure 5. Pairwise abundance ratios for all of the selected proteins are provided in Supplementary Table S2.1 for the respective treatments and fractions. All comparisons were performed separately for each strain (Dv, Df).

**Figure 5:**
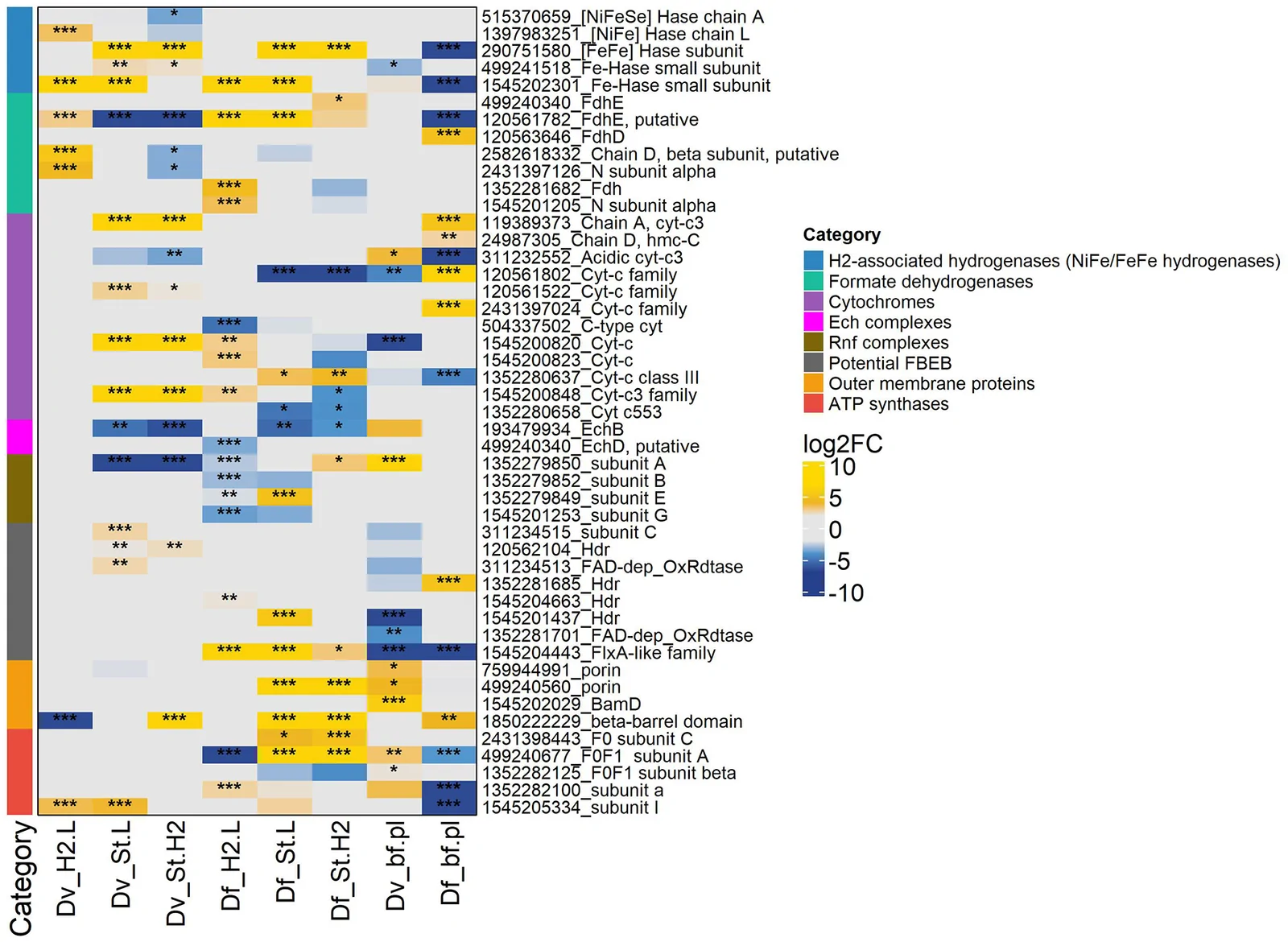
Heatmap showing differential abundances of proteins belonging to categories found relevant to growth with steel and H_2_ (H_2_-associated hydrogenases (NiFe/FeFe hydrogenases); formate dehydrogenases; cytochromes; Ech complexes; Rnf complexes; potential FBEB; outer membrane proteins) additionally along with the category ATP synthases. Each row represents a protein (or protein subunit) with the corresponding accession number. The first six columns represent pairwise comparisons between electron donors (L, H_2_, St) and the last two columns between biofilm (bf) and planktonic fractions (pl) in the steel treatments. Paired electron donors or fractions are separated by a period “.”. Color intensity indicates log_2_(FC) with blue representing decreased abundance (log_2_FC ≤ −2) and yellow indicating increased abundance (log_2_FC ≥ 2). Gray indicates proteins below the selected threshold (−2 ≤ log_2_FC ≤ 2). Only proteins exhibiting at least one significant abundance change across the pairwise comparisons are included. Statistical significance levels of the abundance ratios are indicated (*** for *p*-value ≤ 0.001, ** for *p*-value ≤ 0.01, * for *p*-value ≤ 0.05).

The vast majority of selected proteins (>90%) found in the supernatant were also found in the cellular fractions, including typical membrane-associated proteins (e.g., energy-conserving complexes) as well as cytoplasmic proteins (e.g. those involved in dissimilatory sulfate reduction, Supplementary Tables S2.2A, S2.2B; Table 2). NMDS analysis showed that the supernatant proteome clustered closely with the corresponding cellular (planktonic and biofilm) proteome for the selected proteins (Figure 6), with a similar but a less pronounced pattern observed for all proteins (Supplementary Figure S4). Protein overlap analysis using the whole protein set showed that more than 90% of the proteins detected in the supernatant were also detected in the corresponding cellular fractions. The high percentage of shared proteins which supports that the proteins detected in the supernatant were predominantly derived through cell lysis, rather than representing a true extracellular proteome or active secretion. Therefore, subsequent analyses focused on the planktonic and biofilm cell fractions. NMDS also revealed a close clustering of H_2_ and lactate treatments for both the selected and complete protein datasets of both strains. In contrast, steel treatments were clearly dissimilar for both planktonic and biofilm fractions, with a higher dissimilarity shown by *D. ferrophilus*. Similarities and dissimilarities, however, varied depending on the subset (category) of proteins used for analysis (Supplementary Figures S5–S7). We compared (i) planktonic fractions across the three electron donor conditions, namely H_2_, lactate, and steel (H_2_, L, St), (ii) planktonic and biofilm fractions (pl, bf) of the steel treatments, and (iii) supernatant (su) and cellular (bf, pl) fractions.

**Figure 6:**
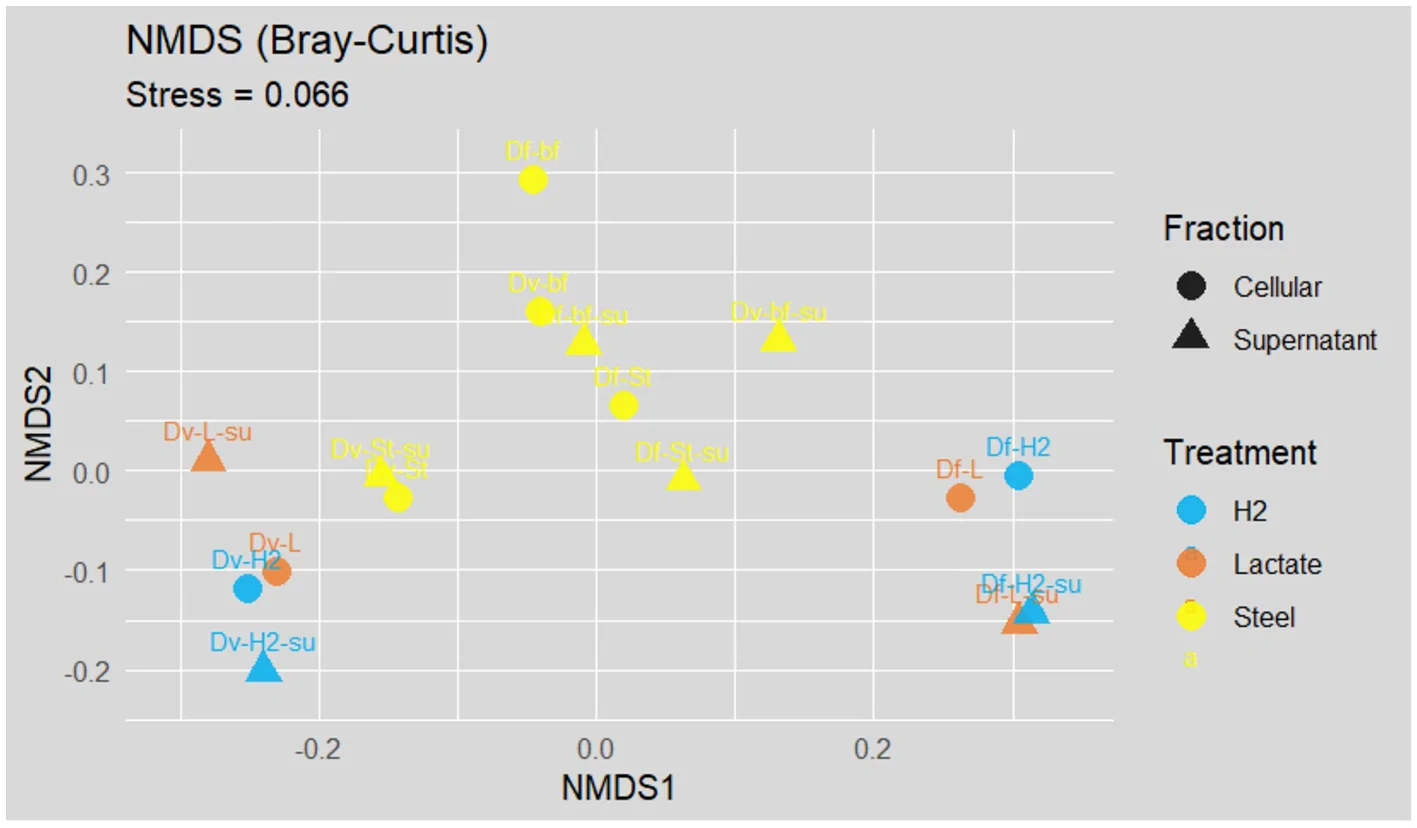
Non-metric multidimensional scaling (NMDS) ordination of selected proteins based on Bray–Curtis dissimilarities (Stress 0.066). Samples are colored for the treatments lactate, H_2_, steel, and symbols indicate the cellular or supernatant fractions.

#### 3.4.2 Proteins involved in energy metabolism with lactate, H_2_ and steel as electron donors

The central cytoplasmic enzymes of dissimilatory sulfate reduction–sulfate adenylyl transferase (Sat), adenylyl-sulfate reductase (AprAB), and dissimilatory sulfite reductase (DsrAB) were detected in all treatments and fractions. Membrane complexes involved in electron transfer, including QmoABC, QrcABCD, DsrMKJOP, units of HmcABCDEF, and TmcD of TmcABCD, were also broadly detected. ATP synthases were significantly more abundant in the H_2_ and steel treatments than in the lactate treatments in both strains (Figure 5 category ATP synthases). The energy-conserving complexes, Rnf, Ech, and Coo, were detected across all treatments and fractions. Ech subunits showed significantly higher abundance in lactate and H_2_ compared to steel treatments in both strains (Figure 5, category Ech complexes). Notably, Ech B were also significantly more abundant in the supernatant fractions of all electron donors in *D.ferrophilus* and only with steel in *D.vulgaris*. Surprisingly, enzymes involved in lactate oxidation to pyruvate and subsequent acetate formation, including lactate dehydrogenase (LDH), pyruvate flavodoxin oxidoreductase (PFOR), phosphate acetyltransferase (PTA) and acetate kinase (ACK), were detected in all treatments and fractions.

Pronounced protein abundance differences were observed for periplasmic hydrogenases (Figure 5 category H_2_-associated hydrogenases ([NiFe]/[FeFe] hydrogenases) and formate dehydrogenases). Soluble periplasmic [NiFe] hydrogenases were significantly more abundant in the H_2_ (Dv-H_2_-pl) and steel (Dv-St-pl) treatments than in the lactate treatment (Dv-L-pl) in *D. vulgaris*. [FeFe] hydrogenases were detected exclusively in the steel treatments of both strains. In addition, a small [FeFe]-hydrogenase subunit was detected only in the H_2_ and steel treatments compared with lactate, in both strains. Formate dehydrogenases were also detected and were significantly more abundant in the H_2_ treatments than in the lactate treatments in both strains (Figure 5 category formate dehydrogenases). In *D. ferrophilus*, formate dehydrogenase was additionally enriched in the steel biofilm treatments (Df-St-bf). The NMDS plot on hydrogenases (Supplementary Figure S6C) reflects these dissimilarities between the electron donors for both strains, and this pattern was more pronounced with *D. ferrophilus*. In supernatant fractions, a nickel-dependent hydrogenase subunit was found to be significantly more abundant for *D. ferrophilus* (Df-St-bf-su vs. Df-St-bf) and a formate dehydrogenase for both strains (Df-St-bf-su vs. Df-St-bf, Dv-L-su vs. Dv-L, Supplementary Table S2.1). Both strains showed a cytochrome-rich proteome, and *c*-type cytochromes were detected across all treatments, including periplasmic cytochrome *c*3, cytochrome *c*553, and cytochrome *c/c*3 family proteins (Figure 5 category cytochromes). Their abundance differed significantly across all pairwise comparisons among lactate, H_2_, and steel treatments in *D. ferrophilus*. The *D. vulgaris* steel treatments (Dv-St-pl) displayed significantly higher abundances of the cytochromes compared to the other two electron donors (Dv-L-pl, Dv-H_2_-pl).

Proteins associated potentially with FBEB including FAD-dependent oxidoreductase, heterodisulfide reductase subunit C, CoB-CoM heterodisulfide reductase, NADH hydrogenase, and FlxA-like protein, were detected across all treatments and fractions. CoB-CoM heterodisulfide reductase was significantly more abundant in *D. vulgaris* steel (Dv-St-pl) compared to lactate (Dv-L-pl) and H_2_ (Dv-H_2_-pl), and in *D. ferrophilus* it was more abundant in H_2_ (Df-H_2_-pl) and steel (Df-St-pl) compared to lactate (Df-L-pl) treatments (Figure 5 category potential FBEB).

#### 3.4.3 Transporters and proteins associated with motility, biofilm formation and possibly corrosion

Proteins related to solute uptake, membrane transport, biofilm formation and regulation, flagellar/motility apparatus as well as additional transporters can be found in Supplementary Tables S2.1 and S2.3, Table 2. L-lactate permeases were detected across treatments and fractions. Porins and sulfate transporters such as SulP were detected ubiquitously. Porins were significantly more abundant in the steel treatments compared to the H_2_ treatments, but not compared to the lactate treatments, in both strains. A few outer membrane proteins showed higher abundance in biofilm compared to planktonic steel fractions in both strains (Figure 5. category outer membrane proteins).

In *D. vulgaris* steel biofilms (Dv-St-bf), porins and BamD were significantly more abundant, whereas in *D. ferrophilus* steel biofilms (Df-St-bf), the outer membrane polysaccharide export protein, TadG family pilus assembly protein, and a β-barrel autotransporter domain-containing protein were more abundant. An outer membrane porin protein and the outer membrane protein assembly factor BamD were significantly more abundant in the *D. ferrophilus* biofilm supernatant fractions (Df-St-bf-su vs. Df-St-bf), while the porin protein was also more abundant in Dv-L-su than in Dv-L (Supplementary Table S2.1; Table 2). The β-barrel autotransporter domain-containing protein was exclusive to the steel treatment (Df-St-pl) compared with the lactate (Df-L-pl) and H_2_ treatments (Df-H_2_-pl) in *D. ferrophilus*. The protein was also exclusive to steel (Dv-St-pl) in *D. vulgaris* and significantly abundant in Dv-H_2_-L compared to Dv-L-pl. An extracellular matrix-associated biofilm protein, hemolysin-type Ca-binding repeat protein (DVU1012), was identified across cellular and supernatant fractions in both strains. NMDS plots show that the transporter protein patterns were almost identical in lactate and H_2_ treatments for both strains, but the planktonic steel and biofilm fractions were dissimilar to each other (Supplementary Figure S5D).

Proteins associated with motility and biofilm formation, including glycosyltransferase and flagellar proteins, were more abundant in biofilm fractions of the steel treatments in both strains (Dv-St-bf, Df-St-bf). Flagellar proteins such as flagellin, hook-basal body, and P-ring components were significantly more abundant. Correspondingly, the NMDS plot supports that biofilm proteomes are dissimilar to those of the planktonic fractions in the lactate, H_2_ and steel treatments (Supplementary Figure S6A). A subset of motility- and cell-surface-associated proteins, including components of flagellar motor (FliL, MotB, FliG, FliN), basal-body/hook (FlgD, FliK), flagellar regulation (FleN) and flagellin-associated proteins (FlaG, flagellins) were detected to be significantly more abundant in the steel supernatants (Dv-St-su vs. Dv-St-pl; Df-St-su vs. Df-St-pl) and biofilm supernatants (Dv-St-bf-su vs. Dv-St-bf; Df-St-bf-su vs. Df-St-bf) of one or the other strain. Notably, FliL and FlgD were also significantly more abundant in Df-L-su compared with Df-L-pl and in Df-H_2_-su compared with Df-H_2_-pl.

## 4 Discussion

### 4.1 Energy metabolism and electron transfer mechanisms

To identify the corrosion mechanisms, and more specifically, the electron-transfer mechanisms adopted by *D. vulgaris* and *D. ferrophilus* when growing with steel, we examined the similarities/dissimilarities in the growth patterns and protein profiles of cells growing with soluble electron donors and compared planktonic and biofilm fractions in steel treatments. Based on our results, we are suggesting four different electron transfer mechanisms (Pathways B, D, E, F, Figure 7), of which two most likely play a role in growth on steel (Pathways E, F). Dissimilatory sulfate reduction (Pathway A, Figure 7) was found to be the central energy-gaining pathway, as biophysicochemical trends and electron balances indicated that sulfate was the main electron acceptor. Accordingly, we found the respective enzymes for sulfate reduction (Sat, AprAB, DsrAB) and transport proteins (SulP), as well as the typical electron-transferring and energy-conserving membrane complexes (QmoABC, QrcABCD and DsrMKJOP) in all samples.

**Figure 7:**
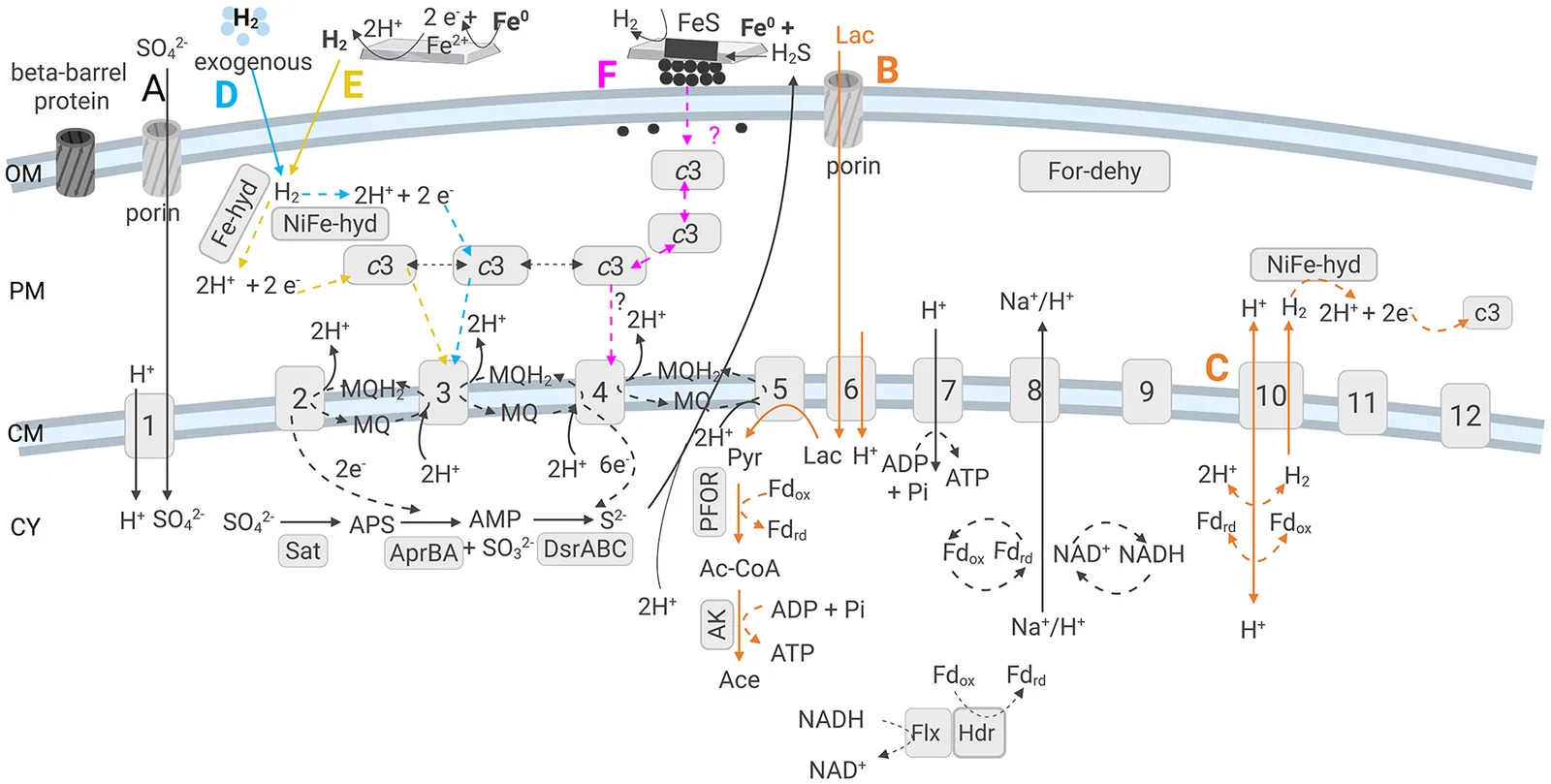
Illustration of energy generation with the three electron-donor conditions: dissimilatory sulfate reduction **(A)**, lactate metabolism **(B)**, H_2_ cycling **(C)**, H_2_ metabolism of exogenously supplied **(D)** and abiotically formed H_2_ on steel (HIMET) **(E)**, and FeS-mediated electron transfer from the steel surface **(F)**. Dashed lines represent proposed electron transfer steps. The numbered boxes indicate the following proteins: SulP (${\text{SO}}_{4}^{2-}$ transporter) (1); QmoABC (2); QrcABCD (3); DsrMKJOP (4); L-Lactate dehydrogenase (5); lactate permease (6); ATP synthase (7); Rnf (8); Coo (9); Ech (10); Tmc (11); and HmcABCDEF (12). Abbreviations: Lac, Lactate; Pyr, Pyruvate; Ace, Acetate; Ni-Fe, Ni-Fe hydrogenase; For-hyd, formate dehydrogenase; Fe-hyd, [FeFe] hydrogenase; NiFe-hyd, [NiFe] hydrogenase; *c*3, *c*-type cytochrome; MQ, menaquinone (oxidized form); MQH_2_ menaquinol (reduced form); sat, ATP-sulfurlylase, AprBA, adenylyl-sulfate reductase; DsrABC, dissimilatory sulfite reductase; OM, outer membrane; PM, periplasmic space; CM, cytoplasmic membrane; CY, cytoplasm. The fold changes of the indicated proteins between the treatments are shown in Fig 5. Created in BioRender. Reddy, L. (2026) https://BioRender.com/fgk1jbq.

#### 4.1.1 Lactate as electron donor

The highest sulfate reduction rates and highest growth yields were obtained with lactate as both an electron donor and organic C source. This was expected, as lactate is a favorable electron donor that supports substrate-level and oxidative phosphorylation (through pyruvate oxidation and H_2_ cycling, Peck, 1966; Barton and Hamilton, 2007; da Silva et al., 2013; Vita et al., 2015). The respective enzymes (LDH, PFOR, PTA and ACK) and lactate transport proteins (LutP) were surprisingly not uniquely abundant in the lactate treatments (Pathway B, Figure 7). This may be because the strains were both pre-cultivated with lactate and although the cells were washed prior to inoculation these proteins may have persisted during the transition in the H_2_ and steel treatments (Lambert and Kussel, 2014).

The energy-conserving hydrogenases EchABCDEF and CooMKLXUHF were identified with all electron donors (Pathway C, Figure 7). With lactate, Ech and Coo may couple ferredoxin oxidation to H_2_ production and energy conservation via membrane-associated electron transfer, whereas with H_2_ they may operate in reverse to regenerate reduced ferredoxin and maintain redox balance (Schölmerich and Müller, 2019). Although genomic studies have reported upregulation of the Ech cluster with H_2_/sulfate and pyruvate/sulfate, and of the Coo cluster with lactate/sulfate (Pereira et al., 2008; Rabus et al., 2015; Schölmerich and Müller, 2019), we detected only a few Ech subunit as significantly more abundant in lactate and H_2_ than in steel, and Coo did not show clear upregulation with lactate. Because Ech and Coo are bidirectional, the similar abundance of these enzymes in lactate and H_2_ treatments may reflect their dual role in ferredoxin-linked redox balancing.

#### 4.1.2 Growing on H_2_ and via HIMET on steel

The second-highest sulfate reduction rates and growth yields were obtained with H_2_ as electron donor. H_2_ can easily diffuse into the periplasm or even into the cytoplasm without the need for transporter proteins (Pathway D showing the H_2_ metabolizes in Figure 7). In terms of biomass with H_2_, and for that matter also with steel as electron donor, additional energy needs to be spent in forming central biosynthetic precursors such as acetyl-CoA and reducing power such as reduced ferredoxin. With steel as the electron donor, it was observed that the majority of cells (97–99%) were planktonic, indicating that direct contact was not needed and that growth was most likely supported by abiotically formed H_2_ (Pathway E in Figure 7). This is also supported by the evidence of consumption of abiotically formed H_2_ when compared to abiotic steel treatments. Though the overall amount of steel was not limiting in our cultures, the formation of H_2_ by steel surface catalysis is a relatively slow process (Massazza et al., 2021; Liu et al., 2023; Reddy et al., 2025) and may be impeded by surface passivation, specifically in the case of *D. ferrophilus*. This would also explain the relatively low sulfate reduction rates and low growth yields in steel treatments. If HIMET was a means of electron transfer, we expected similar energy-metabolizing proteins in steel as in the H_2_ treatments. Respective H_2_ metabolizing periplasmic hydrogenases, e.g., [NiFe] and [FeFe] hydrogenases, were identified for both strains for all electron donors—even for lactate. With lactate, periplasmic hydrogenases are also essential for H_2_ oxidation during energy conservation through H_2_ cycling (Heidelberg et al., 2004; Matias et al., 2005). In general, periplasmic [NiFe]/[FeFe] hydrogenases were expected to be more abundant in the steel and H_2_ treatments, because H_2_ oxidation is a major energy-conservation pathway (Cai et al., 2024). Notably, this pattern was observed for [FeFe] hydrogenases which were exclusively detected in the steel treatments of both strains. This may reflect transient H_2_ formation and localized low-potential electron flux at the steel surface (Deutzmann et al., 2015), which may favor [FeFe] hydrogenases (Peters et al., 2015; Land et al., 2020). However, the more stable H_2_ supply in the H_2_ treatments may better support [NiFe] hydrogenases (Peters et al., 2015). Formate dehydrogenase was significantly more abundant in the H_2_ treatments than in lactate but not compared to the steel treatments in both strains. Previous reports show upregulation of formate dehydrogenase under H_2_/${\text{SO}}_{4}^{2-}$ conditions compared to lactate/${\text{SO}}_{4}^{2-}$ and suggest reversible CO_2_ reduction to formate (Pereira et al., 2008; da Silva et al., 2013) where formate acts as a soluble reservoir of reducing equivalents. Significantly increased abundances of hydrogenases and formate dehydrogenases in the supernatants of *D. ferrophilus* biofilms compared to the corresponding cellular fractions may suggest active secretion. However, a release by extracellular vesicles (Nanninga and Gottschal, 1987; Haynes et al., 1995; Barton and Fauque, 2022) or cell lysis appeared to be more likely, especially as the enrichment of membrane associated proteins maybe reinforced in the application of MWCO filters (10KDa) and acetone precipitation. Extracellular hydrogenase activity has been associated with steel corrosion in methanogens but evidence for such a mechanism in *D. vulgaris* was not found (Deutzmann et al., 2015; Tsurumaru et al., 2018).

The electrons released from H_2_/formate oxidation are transported via periplasmic *c*-type cytochromes (Heidelberg et al., 2004), which were ubiquitously detected. We found significantly higher abundances of *c*-type cytochromes with steel compared to the other two electron donors in *D. vulgaris*. In contrast, in *D. ferrophilus*, many *c*-type cytochrome proteins were more highly abundant across all the electron donors. Overall, it appeared that periplasmic electron transfer was important for growth with each of the three electron donors in order to shuttle periplasmic or probably extracellular electrons to membrane-associated complexes for energy conservation and to be further routed to the cytoplasm. Qrc, Hmc, and Tmc were ubiquitously identified but not specifically more abundant in the H_2_ and steel treatments. The energy-conserving complex, ATP synthase, was more abundant in the H_2_ and steel treatments, consistent with a greater reliance on oxidative phosphorylation than in lactate. Overall, the high abundance of [FeFe] hydrogenases and *c*-type cytochrome in the steel treatments suggests a HIMET mechanism.

Flavin-based electron bifurcation (FBEB) is an important mechanism for regenerating reduced ferredoxin, particularly in organisms growing on H_2_ and via HIMET on steel. In lactate-grown cells, reduced ferredoxin is generated during pyruvate oxidation (Ramos et al., 2015; Vita et al., 2015). Therefore, proteins involved in FBEB were expected to be more abundant in H_2_ and steel compared to lactate treatments. This pattern was partly observed, where CoB-CoM heterodisulfide reductase, a key component of the FlxABCD-HdrABC flavin-based electron bifurcation (FBEB) complex, was significantly more abundant in steel treatments of *D. vulgaris*, and in H_2_ and steel compared to lactate treatments in *D. ferrophilus*.

In contrast to our observations so far, the planktonic steel fractions showed no clear signature of DIMET, such as outer membrane multiheme *c*-type cytochromes. This may not be surprising for the planktonic fractions; therefore, we also investigated the proteome of biofilm cells separately, as discussed in the following chapter.

#### 4.1.3 Biofilm growth and putative DIMET mechanisms

Despite the small proportion of biofilm cells, steel coupons were covered by thick biofilms. Biofilm cells were expected to show protein profiles associated with biofilm formation, regulation, motility and possible surface-associated electron transfer. Accordingly, biofilm-associated proteins were significantly more abundant in biofilm cells than in planktonic cells of both strains. Glycosyl transferases involved in biofilm matrix remodeling were enriched in biofilm cells of both strains. Flagellar proteins are responsible for motility, surface sensing, likely attachment and early biofilm establishment (Haiko and Westerlund-Wikström, 2013; Belas, 2014; Petrova and Sauer, 2016; Pandit et al., 2026). The high abundances suggest enhanced attachment at the steel interface (Liu et al., 2019; Hou et al., 2024). High abundance of TadG family pilus assembly proteins suggests pili-mediated surface adherence and early biofilm formation in *D. ferrophilus* (Li et al., 2019; Evans et al., 2025). Pili may also act as conductive nanowires and have been suggested to facilitate extracellular electron transfer from steel to cell (DIMET) e.g., in *Geobacter sulfurreducens* (Kline et al., 2010; Eaktasang et al., 2013; Deng et al., 2020; Gupta et al., 2020; Lovley and Holmes, 2022; Hou et al., 2024; Liu et al., 2024). However, their direct role in electron transfer remains unconfirmed in *Geobacter species* (Gu et al., 2021; Tang et al., 2021). With respect to DIMET, we did not detect outer membrane multiheme cytochromes (Lahme et al., 2019; Chatterjee et al., 2021; Lekbach et al., 2021) in either species. While porins in *D. vulgaris* and a beta-barrel protein in *D. ferrophilus* were enriched in biofilms, these proteins mainly play a role in nutrient and solute transport across the outer membrane (Hartshorne et al., 2009; Richardson et al., 2012; Zou et al., 2021), rather than shuttling electrons from steel because we did not detect the required redox partners. Significantly increased abundances of porin and BamD in biofilm supernatants of *D. ferrophilus* in addition to flagellar/motility- and cell-surface-associated proteins in the supernatant fractions of either species, were most likely due to their association with the extracellular matrix, potential extracellular vesicles, or cell lysis. The abundances of H_2_-oxidizing periplasmic hydrogenases were not significantly different between the biofilm and planktonic fractions in either strain. In contrast, formate dehydrogenases were more abundant in *D. ferrophilus* biofilms, an observation that may be relevant to formate-mediated electron transfer. Whether this reflects a greater role for formate metabolism at the metal surface remains unclear.

Electron transfer mediated by semiconductive FeS (King and Miller, 1971; Jiang et al., 2014; Murugan et al., 2018; Deng et al., 2020; Hou et al., 2024) may have occurred in both the strains, especially with *D. ferrophilus*. As has been shown by SEM and EDX analysis, Na_2_S-addition and the production of H_2_S by the bacteria led to the formation of very thin flakes and fine threads of FeS on the steel surface. Furthermore, the observation of cells closely adhering to these minerals supports the possibility of FeS-mediated electron transfer as an additional mechanism to HIMET (Pathway F in Figure 7).

### 4.2 Abiotic corrosion reactions influencing growth on steel

In our pure-culture treatments, abiotic processes occurred alongside microbial activity, but corrosion was significantly increased in the presence of the bacteria. H_2_ formation was observed in both saltwater and freshwater abiotic treatments. Here, H_2_ evolution near the metal surface can approach a thermodynamic and kinetic steady state while, in the presence of hydrogen-consuming bacteria, H_2_ partial pressure is kept low, favoring continued H_2_ evolution and increasing corrosion rates (Philips, 2020; Jones et al., 2025). Formate was only detected in abiotic treatments, but an enhanced acetate formation was observed with *D. vulgaris*. Elemental Fe (as well as other transition metals such as Cu, Ni and Mn) can abiotically reduce CO_2_ to formate and acetate via surface-mediated reactions involving H_2_ and C1 intermediates (Varma et al., 2018; Muchowska et al., 2020; Preiner et al., 2020; Heenen et al., 2022; Beyazay et al., 2023; Yang et al., 2023; Mei et al., 2024; Zhao et al., 2026). The enhanced acetate formation with *D. vulgaris* likely reflects a combination of microbial and abiotic carbon reduction on the steel surface. However, we did not find extracellular enzymes that may have been responsible for this process (Lekbach et al., 2021; Mei et al., 2024).

SEM and EDX analysis revealed the complete covering of the steel surface with a thin layer of FeS, resembling mackinawite (Ma et al., 2000) in the abiotic saltwater treatments. In this case, FeS can provide electrical conductivity while simultaneously reducing access to the reactive Fe^0^ surface layer, a process called passivation, and thereby inhibiting H_2_ formation (Enning and Garrelfs, 2014; Qin et al., 2018). This may partly explain the lower yields in *D. ferrophilus* than in *D. vulgaris* on steel. In contrast, discontinuous FeS deposits that do not fully cover the surface, such as those formed during bacterial sulfate reduction, may allow corrosion to proceed via localized H^+^ reduction and at the same time enabling extracellular electron transfer. These observations highlight overall impact of FeS on corrosion processes.

Iron carbonates and phosphates which were formed in both biotic and abiotic treatments are generally considered poorly conductive and may limit electrical contact between the cells and the underlying steel surface (Mercier-Bion et al., 2018). Nonetheless, mineral deposits provided sites for cell attachment and biofilm formation next to a relative reactive steel surface (Donlan and Costerton, 2002; Dang and Lovell, 2016). Besides, carbonate and phosphate anions scavenge the Fe^2+^ forming as siderite (FeCO_3_) and ferrous phosphate minerals which may promote further abiotic corrosion. These minerals can also be responsible for the coprecipitation of sulfate (Zarga et al., 2013; Kartnaller et al., 2018; Reddy et al., 2025). In general, chloride ions can disrupt iron oxide passive films and promote localized corrosion thereby H_2_ formation can occur promoting bacterial growth (Díez-Pérez et al., 2006; Parangusan et al., 2021). Hence, we expected a higher corrosion rate for the saltwater treatments. However, the FeS layer and other abiotic processes may have offset the chloride-driven increase in corrosion, resulting in comparable overall weight loss between freshwater and saltwater treatments.

## 5 Conclusion

In this study biophysicochemical analysis and comparative proteomics were applied to investigate *Desulfovibrio vulgaris* and *Desulfovibrio ferrophilus* with lactate, H_2_ and steel. Microbial activity led to significant steel weight loss compared to abiotic corrosion. Growth with steel supported low biomass and sulfate reduction rates compared to lactate and H_2_. Proteomic evidence revealed high abundances of periplasmic hydrogenases and *c*-type cytochromes with steel as electron donor, similar to the H_2_ treatments, alongside energy conservation through FBEB complex proteins. This together with observed steel growth patterns and abiotic H_2_ formation with steel support H_2_-mediated electron transfer (HIMET) as the primary electron uptake mechanism under our study conditions in both the strains. Iron sulfide precipitates formed with both strains, may provide a conductive interface between cells and the steel surface, supporting the possibility of FeS-mediated electron transfer. The close association of cells with FeS precipitates is in line with this possibility. The low sulfate reduction rates, the relatively small proportion of biofilm cells, the absence of direct cell-to-steel contact and the lack of identified DIMET-associated redox proteins revealed no strong evidence for DIMET under our experimental conditions. Proteomic data reflect steady-state expression and cannot fully exclude transient or localized DIMET activity (e.g., during early biofilm establishment). Overall, our findings improve the understanding of metabolic adaptations of *Desulfovibrio* spp. and their interactions with steel. Both organisms appear to rely primarily on pre-existing hydrogenase-based energy-conserving pathways primarily through HIMET, operating in combination with corrosion-driven abiotic processes such as FeS precipitation. Future studies using conductive atomic force microscopy (c-AFM) or electrochemical impedance spectroscopy (EIS) could directly assess the conductivity and potential electron-transfer properties of FeS precipitates at the cell-steel interface.

Several methodological considerations warrant attention. Shotgun metaproteomics is influenced by proteome coverage, peptide ionization efficiency, database completeness, and sample complexity, which can limit consistent protein detection and complicate interpretation. The substantial overlap in protein abundance across treatments further reflects the multifunctional nature of many enzymes, which may participate in multiple metabolic pathways depending on the available electron donor, complicating assignment to specific metabolic processes. Additionally, protein abundance patterns may be affected by prior growth conditions and acclimatization time and downregulation was not always evident. Future studies should therefore incorporate controlled pre-adaptation experiments to distinguish short-term from long-term physiological responses.

Pure-culture systems provide valuable insights into corrosion under controlled conditions. However, in nature, steel-associated biofilms comprise complex microbial communities in which syntrophic interactions, mineral formation, and dynamic redox gradients may influence the relative importance of different electron transfer pathways and collectively shape corrosion processes. Future studies integrating targeted gene deletion, metaproteomics, metagenomics, and electrochemical analyses will be important for extending mechanistic understanding from simplified laboratory systems to environmentally relevant corrosion environments.

## Data Availability

The protein list generated for this study can be found in the [Figshare] repository [https://doi.org/10.6084/m9.figshare.33005042].
