# Supplementary material for: Comparative proteomics reveals hydrogenase-centered steel-dependent growth and corrosion in Desulfovibrio vulgaris and Desulfovibrio ferrophilus
Source: Front Microbiol. 2026 Sep 22;17:1944645. doi: 10.3389/fmicb.2026.1944645 (PMC13638558; doi:10.3389/fmicb.2026.1944645)
Supplement: Supplementary file 1 [file Supplementary_file_1.docx]

Supplementary Material

# 1 Supplementary Data

## 1.1 H_2_ measurement

For calibration, the anode of the hydrogen sensor was initially polarized to +100 mV against the cathode by connecting it to the Unisense field multimeter. Two-point calibration was performed at 0% sat (ambient air) and 5% sat using a 5% H_2_ (5.0)/95% N_2_ (5.0) gas mixture, which was injected into a 4-ml vials. All values were recorded by the Unisense trace suite software (v3.3).

## 1.2 ddPCR analysis

The PCR reaction mix (final volume= 22 µl) was composed of 11 μl QX 200 ddPCR EvaGreen®Smx (Bio-Rad, Hercules, CA, USA), 7 μl nuclease-free water, 1 μl of forward and reverse primers (4400 nM), and 2 μl of DNA template. The DNA template was diluted in the range of 1:10, 1:100, 1:1000 and 1:10000. 20 µl of the reaction mix was used to generate droplets (Bio-Rad QX200TM Droplet Generator, Bio-Rad, Hercules, CA, USA). The resulting emulsion (40 μl) was transferred to a 96-well plate and sealed. PCR was run in a Bio-Rad C1000-TouchTM Thermal Cycler, Bio-Rad, Hercules, CA, USA. The amplification conditions were as follows: 5 min at 95°C; 50 cycles of 45 s at 96°C, 1 min at 57°C, and 1 min at 72°C (ramp rate 1°C s^-1^). Enzymes were deactivated by a 5 min hold at 4°C, followed by a 5 min hold at 90°C. Each run included a non-template control (nuclease-free water) and a positive control (1:1000 diluted gDNA of *D. vulgaris* (DSM 2119) strain). Droplets were read on a Bio-Rad QX200 Droplet Reader, Bio-Rad, Hercules, CA, USA. Only samples with at least 11,000 accepted droplets were used for further analysis. The positive droplet threshold was set manually using the QuantaSoft^TM^ Analysis Pro software (Bio-Rad, Hercules, CA).

## 1.3 Bottom-up proteomics

### 1.3.1 *Protein enrichment and purification from supernatant sample*

The workflow of the protein preparation is indicated in Table S2. Batches of 15 ml or 0.5 ml supernatant samples were transferred to 10 kDa Amnicon centrifugal filters 15 ml and 0.5 ml, respectively. To determine the retentate volume, each component of the device- cap, filter and centrifuge tube was weighed before and after sample application, before centrifugation, as well as after centrifugation. The retained fraction was then subjected to ice-cold acetone precipitation to remove salts from the medium and enrich the protein fraction. (Baghalabadi and Doucette, 2020). The concentrated protein pellets were resolubilized in 100 µl of SDT buffer and subjected to reduction, alkylation and tryptic digestion through SP3-beads.

### 1.3.2 *Reduction, alkylation, tryptic digestion and peptide clean-up using SP3-beads*

Protein concentrations of cell lysates from planktonic and biofilm cultures and enriched, purified supernatant protein fractions were estimated using the reducing-agent-compatible Pierce BCA Protein Assay Kit (Fisher Scientific, Schwerte, Germany). Details of the SP3 bead-based protein clean-up procedure are described in Hughes et al. (2019) and Mikulášek (2021).

For each sample, 30 – 60 µl was subjected to reduction with DTT (5 mM final concentration) followed by alkylation (100 mM final concentration). Alkylated proteins were bound to SP3 beads at 20 µg µl^-1^. After binding, the beads were washed five times with ethanol to remove contaminants and salt. Proteolytic cleavage was performed on-bead using trypsin (50 ng µl^-1^). The peptide lysates were eluted with 60 µl of 50 mM NH_4_HCO_3_ solution, which was repeated twice, and the eluted peptides were pooled.

Peptide concentration was determined using the Pierce Fluorometric Peptide Assay with a working range of 5 – 1000 µg ml^-1^ (Fisher Scientific, Schwerte, Germany). The peptide lysates were lyophilized by vacuum centrifugation (RVC 2-18 CD Plus, Christ, Osterode am Harz, Germany). The dried lyophilized pellets were resuspended in 0.1% formic acid and subjected to C_18_-desalting. Desalted peptide lysates were again lyophilized and resuspended in 0.1% formic acid for nanoLC-MS/MS analysis.

Before injection, peptide concentrations were additionally checked by measuring the absorbance at 280 nm using a NanoDrop (NanoDrop 2000/2000c spectrophotometer, Thermo Fisher Scientific, Wilmington, USA).

### 1.3.3 *Peptide lysate analysis via nanoUHPLC-HRMS/MS*

Peptide lysates of ~1 µg were injected on a trapping column (Acclaim PepMap 100 C_18_, 3 μm, nanoViper, 75 μm x 2 cm, Thermo Fisher Scientific) at 5 μl min^–1^ using 2% ACN with trifluoroacetic acid (0.08%). Peptide separation was achieved using an analytical column (Acclaim PepMap 100 C_18_, 3 μm, nanoViper, 75 μm x 25 cm, Thermo Fisher Scientific) and gradient elution at a flow rate of 300 μl min^-1^. The mobile phase consisted of 0.1% formic acid in ultrapure water (solvent A) and 80% ACN/ 0.1% formic acid in a mixture of 80 % ACN and 20 % ultrapure water (solvent B). The gradient was as follows: 4% B to 55% B in 125 min. Full MS spectra (350 – 1,550 *m/z*) were acquired with a resolution of 120,000 and an automatic gain control (AGC) target value of 3 × 10^6^ ions.

# 2 Supplementary Figures and Tables

## 2.1 Supplementary Figures

## Supplementary Figure S1. Time trends of relevant biophysicochemical parameters: pH (A, B) and Fe (II) solids (C) in the biotic treatments of both strains monitored over 30 days of incubation.

## Supplementary Figure S2. Time trends of relevant biophysicochemical parameters: Fe(II) solids (A), change in sulfate (B) and sulfide (C, D) concentrations in freshwater and saltwater abiotic treatments monitored over 30 days of incubation.

**HCl- cleaned steel platelets**

**Freshwater**

**Salt**

**10 µm**

**(A)**

**(C)**

**craters**

**1 µm**

**100 nm**

**(C)**

**(C (i))**

**1 µm**

**(D)**

**(E)**

**(G)**

**10 µm**

**(G)**

**layered crater with needles**

**G (i)**

**1 µm**

**100 nm**

**(F)**

**10 µm**

**(H)**

**(H)**

**5 µm**

**100 nm**

**(B)**

**10 µm**

## Supplementary Figure S3. SEM images of the cleaned steel platelets after 4 weeks of incubation with *D. vulgaris* culture (A, C) and *D. ferrophilus* (B, D). Abiotic control platelets: freshwater (E, G) and salt (F, H).

Supplementary Figure S4. Non-metric multidimensional scaling (NMDS) ordination of the complete proteome based on Bray–Curtis dissimilarities (Stress 0.044). Samples are coloured for the treatments lactate, H_2_, steel, and biofilm, and symbols indicate the planktonic or supernatant fractions. Distances between samples represent differences in overall proteome composition.

Supplementary Figure S5. NMDS ordination of cellular protein abundance profiles (planktonic and biofilm) for the functional categories/modules: complete proteins (A), lactate metabolism (B), FBEB mechanism (C) and other transporters (D). Samples are coloured for the treatments and fraction (lactate, H₂, steel, and biofilm) and shaped according to the strains (*D.vulgaris* and *D.ferrophilus*). Ordinations were based on Bray–Curtis dissimilarities, and stress values are indicated in each panel.

Supplementary Figure S6. NMDS ordination of the cellular protein abundance profiles (planktonic and biofilm) for the functional categories/modules: biofilm proteins (A), electron carriers (B), hydrogenases (C) and membrane complexes (D). Samples are coloured by treatment and fraction (lactate, H₂, steel, and biofilm) and shaped according to the strains (*D.vulgaris* and *D.ferrophilus*). Ordinations were based on Bray–Curtis dissimilarities, and stress values are indicated in each panel.

**(A)**

**(B)**

Supplementary Figure S7. NMDS ordination of the cellular protein abundance profiles (planktonic and biofilm) for the functional categories/modules: ATP synthase (A) and cytochromes (B). Samples are coloured by treatment and fraction (lactate, H₂, steel, and biofilm) and shaped according to the strains (*D.vulgaris* and *D.ferrophilus*). Ordinations were based on Bray–Curtis dissimilarities, and stress values are indicated in each panel.

## 2.2 Supplementary Tables

## Supplementary Table S1. Freshwater medium composition.

| **Freshwater medium** | **ml l^-1^** | **g l^-1^** | **mM** |
| --- | --- | --- | --- |
| NH_4_Cl |  | 0.267 | 5 |
| KH_2_PO_4_ |  | 0.204 | 1.5 |
| KCl |  | 0.447 | 6 |
| NaCl |  | 0.234 | 4 |
| CaCl_2_*2H_2_O |  | 0.147 | 1 |
| MgCl_2_*6H_2_O |  | 0.305 | 1.5 |
| MgSO_4_*7H_2_O |  | 0.123 | 0.5 |
| Vitamin solution* | 1 |  |  |
| Vitamin B12 solution* | 1 |  |  |
| Thiamine solution* | 1 |  |  |
| Se-W-solution* | 1 |  |  |
| NaHCO_3_ | 30 |  | 30 |
| Na_2_SO_4_ | 20 |  | 20 |

*These solutions were prepared according to Widdel and Bak (1992)

A total of 1 l of media was prepared after adding all the solutions (including lactate or acetate) by making up the media with autoclaved Milli-Q water.

Supplementary Table S2. Summary of the protein preparation from source up to peptide measurements.

| Sample matrix | Planktonic liquid sample (100 ml of each replicate) | | Biofilm on steel coupon (1.5 ml per coupon) | |
| --- | --- | --- | --- | --- |
| Pre-processing | Centrifugation at 4,863 x *g* for 30 min at 20 °C | | | |
| Types of fractions | Planktonic cell pellet | Culture liquid supernatant | Biofilm cell pellet | Biofilm supernatant |
| Cellular components and probable protein type | Cytoplasmic, membrane proteins and extracellular vesicles | Extracellular, lysed and soluble proteins | Cytoplasmic and membrane proteins | Extracellular, lysed and soluble proteins in EPS and extracellular vesicles |
| Cell concentration | Centrifugation at 18,000 x *g*, 4 °C, 15 min |  | Centrifugation at 18,000 x g, 4 °C, 15 min |  |
| Cell lysis | Cell lysis in 1 % SDT buffer |  | Cell lysis in 1 % SDT buffer |  |
| Protein enrichment |  | Sample enrichment by 10 kDa MWCO filters (15 ml and 0.5 ml) |  | Sample enrichment by 10 K MWCO filters (0.5 ml) |
| Protein purification |  | Acetone precipitation |  | Acetone precipitation |
| Protein quantification | BCA assay (reducing agent compatible) | | | |
| Protein preparation | Reduction, alkylation and SP3 binding, clean-up and overnight tryptic digestion at 37°C | | | |
| Peptide elution | Elution of peptides in 50 mM NH_4_HCO_3_ solution | | | |
| Peptide concentrating | Lyophilization of the peptides and solubilization in 0.1% formic acid | | | |
| Peptide purification | - C_18_ ZipTip purification - Lyophilization of the de-salted peptide lysates | | | |
| Peptide quantification and measurement | - Resolubilization in 0.1% formic acid - NanoDrop (absorbance at 280 nm) - LC/MS measurement | | | |

Supplementary Table S3: Proteomics sample names (refer to complete protein list available at <https://figshare.com/s/04f3750c083996ec803a>).

| **Proteomics sample names** | **Treatment abbreviations** |
| --- | --- |
| 1 - 3 | Dv-L-pl |
| 4 - 6 | Dv-H_2_-pl |
| 7 - 9 | Dv-St-pl |
| 10 - 12 | Df-L-pl |
| 13 - 15 | Df-H_2_-pl |
| 16 - 18 | Df-St-pl |
| Pm7 - pm9 | Dv-St-bf |
| Pm16 - Pm18 | Df-St-bf |
| US1 - US3 | Dv-L-su |
| US4 - US6 | Dv-H_2_-su |
| US7 - US9 | Dv-St-su |
| US10 - US12 | Df-L-su |
| US13 - US15 | Df-H_2_-su |
| US16 - US18 | Df-St-su |
| USpm1 - Uspm3 | Dv-St-bf-su |
| USpm4 – Uspm6 | Df-St-bf-su |

**Supplementary Table S4**. Average growth parameter values with lactate, H_2_ and steel as electron donors in the biotic treatments. Due to the variable growth observed with the *D. ferrophilus* lactate treatment, only one of the replicate values is depicted (asterisk symbol).

| Parameters | Dv-L | Dv-H_2_ | Dv-St | Df-L | Df-H_2_ | Df-St |
| --- | --- | --- | --- | --- | --- | --- |
| Yield coefficients (mg protein mmol^–1^ SO_4_^2–^ reduced) | 21.75 ± 0.66 | 6.01 ± 0.68 | 4.68 ± 0.62 | 22.10* | 22.58 ± 3.37 | 1.74 ± 0.34 |
| Yield (gene copies mmol^–1^ SO_4_ ^2–^ reduced) | 32.53 ± 7.64 | 26.85 ± 23.89 | 28.55 ± 8.20 | 37.35* | 32.50 ± 18.31 | 6.18 ± 2.00 |

**Supplementary Table S5**. Cumulative Na_2_S addition to saltwater treatments.

| Abiotic treatments – Adding 5 ml of 1.70 mM Na_2_S to 80 ml medium per sampling | | | | | | | | |
| --- | --- | --- | --- | --- | --- | --- | --- | --- |
| Day | 0 | - | - | - | 16 | 23 | 27 | 31 |
| mM | 1.70 | - | - | - | 1.80 | 1.90 | 2.00 | 2.10 |
| Biotic treatments – Adding 5 ml of 1.70 mM Na_2_S to 100 ml medium per sampling | | | | | | | | |
| Day | 1 | 3 | 8 | 11 | 18 | 24 | 26 | 31 |
| mM | 1.70 | 1.78 | 1.86 | 1.94 | 2.02 | 2.10 | 2.18 | 2.25 |

**Supplementary table S6.** Representation of the stoichiometric calculations of Na_2_S addition and contribution to steel weight loss in the saltwater (biotic and abiotic) treatments.

| Treatments | Steel (Fe^0^) coupon weight [T0] | Steel (Fe^0^) coupon weight [Tend] | Total Fe^0^ weight loss (g) [T0-Tend] | Total Fe^0^ weight loss (mg/cm2) | Total Fe^0^ weight loss (mM) | Avg | St.dev | Average electron release (mM) | Total saltwater Fe^0^ weight loss (mM) – [total Na_2_S accumulation]  (2. 1 mM for abiotic and 2.25 mM for biotic treatments) | Avg | St.dev | Average electron release after Na₂S subtraction (mM) |
| --- | --- | --- | --- | --- | --- | --- | --- | --- | --- | --- | --- | --- |
| Dv-St-a | 13.23 | 13.21 | 0.03 | 1.93 | 4.80 | 5.09 | 0.26 | 10.18 | N.A. | N.A. | N.A. | N.A. |
| Dv-St-b | 13.13 | 13.10 | 0.03 | 2.09 | 5.19 |  |  |  | N.A. |  |  |  |
| Dv-St-c | 13.16 | 13.13 | 0.03 | 2.12 | 5.28 |  |  |  | N.A. |  |  |  |
| Df-St-a | 13.13 | 13.08 | 0.05 | 3.91 | 9.74 | 8.77 | 1.26 | 17.54 | 7.49 | 6.52 | 1.26 | 13.04 |
| Df-St-b | 13.14 | 13.09 | 0.05 | 3.71 | 9.22 |  |  |  | 6.97 |  |  |  |
| Df-St-c | 13.14 | 13.10 | 0.04 | 2.95 | 7.34 |  |  |  | 5.09 |  |  |  |
| Ab-fresh-a | 13.15 | 13.14 | 0.01 | 0.97 | 3.29 | 3.36 | 0.93 | 6.72 | N.A. | N.A. | N.A. | N.A. |
| Ab-fresh-b | 13.14 | 13.12 | 0.02 | 1.28 | 4.32 |  |  |  | N.A. |  |  |  |
| Ab-fresh-c | 13.12 | 13.11 | 0.01 | 0.73 | 2.46 |  |  |  | N.A. |  |  |  |
| Ab-salt-a | 13.17 | 13.15 | 0.02 | 1.11 | 3.76 | 3.70 | 0.06 | 7.40 | 1.66 | 1.60 | 0.06 | 3.20 |
| Ab-salt-b | 13.13 | 13.11 | 0.02 | 1.08 | 3.65 |  |  |  | 1.55 |  |  |  |
| Ab-salt-c | 13.13 | 13.11 | 0.02 | 1.09 | 3.69 |  |  |  | 1.59 |  |  |  |
